# A Mechanotransduction-Aware Strategy for Enhancing MSC Potency via 3D Culture and Localized Delivery

**DOI:** 10.34133/cbsystems.0552

**Published:** 2026-03-24

**Authors:** Xuyu Gu, Jijun Sun, Yifei Zhou, Dongning Lu, Weixi Wang, Cong Ye

**Affiliations:** ^1^ Department of Oncology, Shanghai Pulmonary Hospital, Tongji University, Shanghai 200433, China.; ^2^ Department of Thoracic Surgery, Shanghai Pulmonary Hospital, Tongji University, Shanghai 200433, China.; ^3^ Department of Geriatrics, Zhongshan Hospital, Fudan University, Shanghai 200032, China.

## Abstract

Acute lung injury (ALI) is characterized by uncontrolled inflammation, oxidative stress, and fibrotic remodeling, yet mesenchymal stem cell (MSC) therapies remain limited by poor retention and insufficient microenvironmental adaptation. Here, we engineered a composite system, GelMA@hMSCs-Alg-RGD (hereafter referred to as the sandwich composite), in which RGD (Arg-Gly-Asp) -functionalized alginate microbeads support human MSCs and are encapsulated within an adhesive, stress-relaxing dopamine-modified GelMA (GelMA-DA) hydrogel. This design provided a 3-dimensional low-tension niche that preserved MSC identity while enhancing paracrine potency, antioxidative capacity, and resistance to apoptosis. Conditioned media from hMSCs-Alg-RGD promoted endothelial proliferation, migration, invasion, and tube formation, while attenuating oxidative stress in a partially cytoskeleton-dependent manner. In fibroblasts, treatment suppressed alpha-smooth muscle actin stress fiber formation, focal adhesion maturation, and Yes-associated protein nuclear translocation, thereby preventing myofibroblast differentiation and restoring isotropic morphology. GelMA@hMSCs-Alg-RGD enabled rapid gelation, robust wet adhesion, stress-relaxing mechanics, and controlled degradation, resulting in prolonged pulmonary retention confirmed by in vivo and ex vivo imaging. In murine ALI, this strategy alleviated edema, reduced inflammatory cytokines (interleukin-6, tumor necrosis factor-α, and interleukin-1β), myeloperoxidase activity, and lipid peroxidation (malondialdehyde), while enhancing superoxide dismutase activity, improving survival, and reshaping the immune microenvironment through reduced neutrophil infiltration and enhanced macrophage M1 → M2 polarization. Together, these results establish GelMA@hMSCs-Alg-RGD as a bioengineered therapeutic that integrates localized retention with paracrine amplification to reprogram immune and mechanical microenvironments, offering a broadly applicable platform for MSC-based regenerative medicine.

## Introduction

Acute and chronic lung injuries—from chemical, infectious, or surgical insults to progressive fibrotic remodeling—remain leading causes of morbidity and mortality [1]. In acute settings such as acute lung injury (ALI) and acute respiratory distress syndrome (ARDS), diffuse inflammation and barrier failure rapidly compromise gas exchange; in subacute and chronic disease, maladaptive repair and fibroblast–myofibroblast activation culminate in matrix accumulation, tissue stiffening, and loss of compliance [2–4]. Clinically, management of ARDS is still largely supportive, centered on lung-protective ventilation, judicious positive end-expiratory pressure (PEEP)/oxygenation strategies, prone positioning for appropriate patients, fluid management, and rescue measures such as extracorporeal membrane oxygenation (ECMO) when needed. Despite advances in ventilation strategies and anti-inflammatory care, current therapies seldom address 3 features that are intrinsic to the lung as an organ: (a) the spatial heterogeneity of injury (focal leaks, patchy inflammation, and local fibrotic foci); (b) the cyclical mechanical load imposed by respiration; and (c) the multicellular crosstalk among stromal, immune, and endothelial compartments that governs resolution versus progression [5]. These gaps motivate localized, mechanism-aware interventions that can both stay where they are needed and shape cell signaling toward pro-resolution outcomes [6,7].

Mesenchymal stem cells (MSCs) are attractive for pulmonary repair because they secrete trophic mediators, release extracellular vesicles (EVs), and modulate macrophage and fibroblast behavior through paracrine and juxtacrine pathways [8]. Yet, clinical translation has been limited by coupled bottlenecks [9]. First, retention is poor: cells delivered intratracheally or topically are rapidly cleared or redistributed, especially on wet, surfactant-coated, and moving surfaces [10]. Second, potency is context-dependent: MSCs expanded and administered as 2-dimensional (2D) suspensions tend to lose the low-tension, immunomodulatory phenotype observed in 3-dimensional (3D) microenvironments, where cytoskeletal tone and metabolic programs are distinct [11]. Third, delivery matrices lag behind need: few materials can adhere conformally to wet, compliant, and continuously deforming lung tissues while remaining thin enough to preserve ventilation and soft enough not to impose pathological tension on resident or therapeutic cells [12]. An enabling platform must therefore couple on-site residence with biophysical correctness—maintaining survival cues without forcing high focal-adhesion maturation or Yes-associated protein (YAP)/transcriptional coactivator with PDZ-binding motif (TAZ)-driven profibrotic signaling [13].

Biomaterials offer a principled route to meet these constraints if designed around 3 levers. (a) Ligand presentation. Integrin-engaging motifs such as RGD, displayed at controlled density, support cell survival and moderate adhesion while avoiding the excessive cytoskeletal tension that blunts immunomodulation or biases lineage; (b) Mechanics and dissipation [9]. Soft, stress-relaxing networks lower actomyosin tone and attenuate the adhesion–contractility axis that feeds myofibroblast transition; viscoelastic dissipation is particularly important under cyclic stretch; (c) Wet adhesion and form factor. A thin, rapidly formable overcoat that bonds in water and tolerates blood/surfactant contamination can immobilize carriers at the lesion, distribute load, and preserve local compliance during breathing. RGD-functionalized alginate microspheres have been extensively explored as 3D carriers for human mesenchymal stem cells (hMSCs), improving cell survival, adhesion/spreading, and paracrine activity in a range of tissue-repair settings, and have been summarized in recent reviews. However, translating such microbead-based delivery to the lung faces a distinct and under-addressed barrier: the pulmonary lesion surface is moist, highly dynamic, and continuously subjected to respiratory motion and fluid flushing, which can rapidly displace transplanted cells/microcarriers and limit effective local exposure. Here, rather than revalidating the microbead platform itself, we introduce a wet-adhesive “sandwich” strategy in which a rapidly in situ-curable, stress-relaxing hydrogel layer anchors hMSCs-Alg-RGD microbeads to the lung surface, thereby prolonging local residence and enabling a more direct linkage between retention, clinically relevant functional recovery, and longer-term remodeling outcomes [14,15].

Here, we introduce a modular strategy that integrates these design rules: RGD-functionalized alginate microbeads (Alg-RGD μS) as 3D niches for MSCs (hMSCs-Alg-RGD), combined with a catechol-modified gelatin (Gel-DA) wet-adhesive overcoat applied as a thin “sandwich” film [16,17]. Alginate is clinically familiar, cytocompatible, and readily fabricated into uniform microbeads with a large specific surface area for mass transport. By grafting RGD at defined densities, the microbeads provide controlled integrin engagement that stabilizes MSC attachment while permitting low-to-moderate cytoskeletal tension. The microbead geometry preserves clustered, quasi-3D morphologies linked to enhanced oxidative-stress tolerance and paracrine output compared with 2D monolayers [18]. Gel-DA contributes catechol chemistry for underwater adhesion to tissue and hydrated polymers and can be formulated to gel within ~1 to 3 min while remaining soft and stress-relaxing [19]. As a composite—GelMA/MSC@Alg-RGD—the construct spreads as a submillimeter film over irregular, wet substrates, immobilizes microbeads against respiratory motion, and preserves local compliance, addressing both residence and mechanical matching [20].

**Fig. 1.**
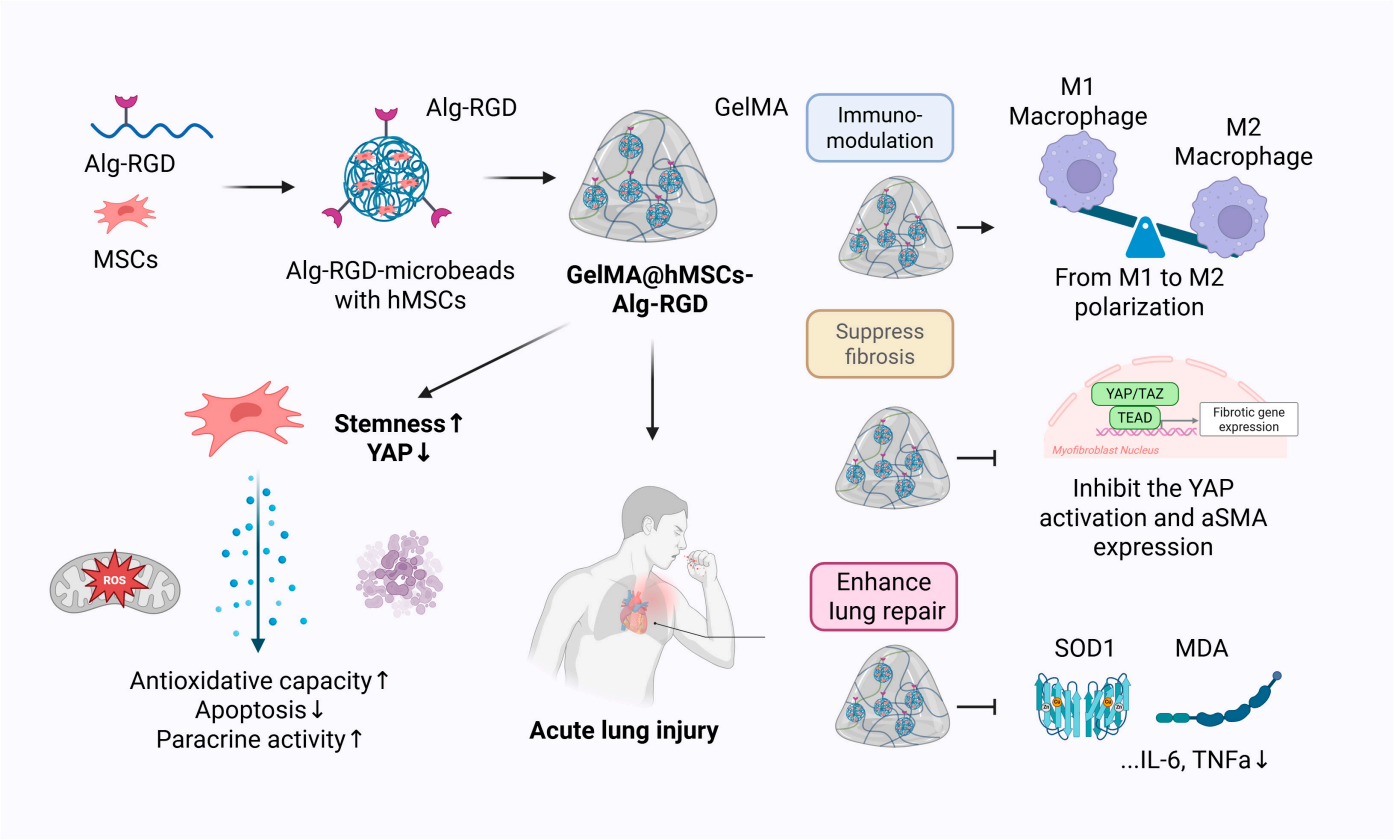
Schematic illustration of the preparation of GelMA@hMSCs-Alg-RGD and its therapeutic application in lung injury.

This study advances a broader concept: cells are only as potent as the microenvironments that host them. Rather than treating MSCs as stand-alone drugs, we design a retention-competent, stress-aware microenvironment that keeps cells where biology is needed and keeps their signaling in a therapeutic lane. By integrating controlled ligand presentation, compliant viscoelastic mechanics, and robust wet adhesion into a single minimally invasive format, the platform addresses key barriers to pulmonary cell delivery—rapid washout/short residence on a moist, dynamically moving surface and the resulting loss of effective local exposure—while preserving 3D microenvironmental support for MSC function (Fig. 1). Specifically, hMSCs are surface-seeded onto preformed Alg-RGD microbeads, and the resulting bead–cell complexes are locally anchored to the injured lung surface using a wet-adhesive hydrogel “sandwich” layer that maintains interfacial stability during breathing. Built on familiar, tunable, and scalable biomaterials (alginate and gelatin), the approach is designed with practical manufacturability in mind. In the sections that follow, we detail the fabrication and multiscale characterization of GelMA@hMSCs-Alg-RGD, quantify improvements in local retention and functional outputs relative to 2D delivery, examine how these changes attenuate fibroblast activation under inflammatory challenge, and demonstrate tissue-level benefits after localized application to injured lung.

## Materials and Methods

### Materials and reagents

Sodium alginate (low viscosity; NovaMatrix, Norway), RGD peptide (Gly-Arg-Gly-Asp-Ser, >95%; GL Biochem [Shanghai] Ltd., China), 1-ethyl-3-(3-dimethylaminopropyl)carbodiimide hydrochloride (EDC·HCl; Sigma-Aldrich, USA), *N*-hydroxysuccinimide (NHS; Sigma-Aldrich, USA), 2-(*N*-morpholino)ethanesulfonic acid (MES; Sigma-Aldrich, USA), Tris base (Sigma-Aldrich, USA), calcium carbonate (CaCO₃; Sigma-Aldrich, USA), glucono-δ-lactone (GDL; Sigma-Aldrich, USA), gelatin type A from porcine skin (Sigma-Aldrich, USA), and ascorbic acid (Sigma-Aldrich, USA) were used in the study. β-D-mannuronic acid lactone and other specialty chemicals used in this study were obtained from Aladdin Bio-Chem Technology (China). Dimethyl sulfoxide was purchased from Sigma-Aldrich (USA). Phosphate-buffered saline (PBS), low-glucose Dulbecco’s Modified Eagle Medium (DMEM), TrypLE Express, fetal bovine serum (FBS), penicillin/streptomycin, L-glutamine, and nonessential amino acids were purchased from Thermo Fisher Scientific (USA; Gibco). Endothelial growth medium-2 (EGM-2) was purchased from Lonza (USA). Matrigel basement membrane matrix and Transwell inserts (8 μm) were purchased from Corning (USA). Annexin V/PI apoptosis detection kit was purchased from BD Biosciences (USA). Calcein-AM/PI live/dead staining reagents were purchased from Yeasen Biotechnology (China). 5-Ethynyl-2′-deoxyuridine (EdU) proliferation assay (Click-iT) and TRIzol reagent were purchased from Invitrogen (USA). DCFH-DA and Cell Counting Kit-8 (CCK-8) were purchased from Beyotime Biotechnology (China). 4′,6-Diamidino-2-phenylindole (DAPI) was purchased from Sigma-Aldrich (USA). Crystal violet and lipopolysaccharide (LPS, *Escherichia coli*) were purchased from Sigma-Aldrich (USA). Mitomycin C and small-molecule inhibitors (Y-27632, blebbistatin, and latrunculin A) were purchased from Selleck Chemicals (USA). Phalloidin-TRITC was purchased from Invitrogen (USA). Primary antibodies against alpha-smooth muscle actin (α-SMA), vinculin, YAP, pFAK(Y397), and pMLC were purchased from Cell Signaling Technology (USA) or Abcam (UK), and flow-cytometry antibodies (CD90, CD105, CD11b, CD19, CD31, and CD34) were purchased from BD Biosciences (USA). Unless otherwise stated, all reagents were of analytical grade.

### Synthesis and characterization of Alg-RGD

Because 1.5% (w/v) alginate solutions are highly viscous, coupling reagents were introduced as predissolved concentrated stocks to avoid local over-concentration and lump formation. Briefly, alginate (1.5% w/v) was fully hydrated overnight and degassed, then equilibrated in 0.1 M MES buffer. The reaction pH was adjusted and maintained at pH 5.5, which is optimal for EDC/NHS activation and coupling. EDC·HCl and NHS were freshly dissolved in the same buffer and added dropwise to the alginate solution under continuous stirring to reach final concentrations of 50 mM EDC and 25 mM NHS (EDC:NHS molar ratio = 2:1). After 15 min of preactivation, the RGD peptide stock solution (10 mg/ml in MES buffer) was added dropwise to a final concentration of 1.0 mg/ml, and the reaction was allowed to proceed for 12 h at 25 °C while maintaining pH within 5.5. After completion, the product was extensively dialyzed (molecular weight cutoff, 3.5 kDa) against deionized water for 48 h at 4 °C with frequent water changes (every 6 h) to remove unreacted peptide and small molecules before subsequent microbead fabrication. After completion, the product was extensively dialyzed to remove unreacted peptide and small molecules before subsequent microbead fabrication.

The degree of RGD grafting was estimated using 2,4,6-trinitrobenzene sulfonic acid, with a target of 0.5 to 2.0 mol% (relative to alginate sugar units).

Characterization: Fourier transform infrared spectroscopy (Nicolet iS10, Thermo Fisher Scientific) and H nuclear magnetic resonance spectroscopy (AVANCE III 400 MHz, Bruker BioSpin) were used to confirm RGD grafting. ζ-potential was measured using a Zetasizer Nano ZS90 (Malvern Panalytical).

### Preparation of Alg-RGD microspheres

A 1.0% to 1.2% Alg-RGD aqueous solution containing 0.4 to 0.6 equivalents of CaCO₃ (relative to -COO^-^ groups) was ultrasonically dispersed. This solution was then emulsified by dripping it into a paraffin oil phase containing 0.5% to 1.0% v/v Span 80 at 1,000 to 1,500 rpm for 5 to 10 min. Gelation was triggered by adding GDL at an equivalent molar amount, and the reaction proceeded for 30 to 45 min. The emulsion was broken with isopropanol, and the microspheres were washed with PBS and collected using 70/40-μm sieves, with a target size of 50 to 120 μm (*d*_50_).

The dispersed phase consisted of 1.0% Alg-RGD with 0.5 equivalents of CaCO_3_, while the continuous phase was 2% poly(vinyl alcohol). The droplets were immediately crosslinked upon collection in a GDL-containing solution.

The sphericity and particle size were measured using bright-field microscopy. Laser particle sizing was performed when necessary. Time-dependent rheology was used to record the storage modulus (*G*′), loss modulus (*G″*), and gelation point.

### Preparation and characterization of gel-DA (wet-adhesive film)

Dopamine was grafted onto GelMA via carbodiimide-mediated amidation. Briefly, the carboxyl groups on GelMA were activated using EDC (optionally with NHS to improve coupling efficiency), followed by reaction with dopamine hydrochloride to obtain dopamine-modified GelMA (GelMA-DA). The reaction was carried out under mildly acidic to neutral conditions (typically pH ~5.5 to 7.0) to preserve catechol functionality. The resulting GelMA-DA was purified by extensive dialysis against deionized water to remove residual reagents and salts, and then lyophilized for storage.

For hydrogel preparation, GelMA-DA was dissolved in sterile PBS to a final concentration of 6% to 8% (w/v) and mixed with a photoinitiator (Irgacure 2959 0.1% w/v). Gelation was achieved by light-triggered photopolymerization of methacrylate groups under defined irradiation conditions (365 nm UV, 5 mW/cm^2^ for ~60 s).

Rheological characterization was performed using small-amplitude oscillatory shear. Time and frequency sweeps were conducted at 1% strain (within the linear viscoelastic region) to record storage (*G*′) and loss (*G*″) moduli. Stress relaxation was measured by applying a step strain of 5% to 10% and recording the decay of stress over time; relaxation time constant(s) (*τ*) were obtained by fitting the relaxation curves as described in the "Statistical analysis" section.

### Cell culture and 3D encapsulation

Human MSCs from a compliant tissue bank were used. Cells were cultured in low-glucose DMEM supplemented with 10% MSC-qualified FBS, 1% P/S, and 2 mM L-Gln. Human umbilical vein endothelial cells (HUVECs) were cultured in EGM-2 complete medium. Human pulmonary fibroblasts (HPF or MRC-5) were cultured in DMEM with 10% FBS. Alg-RGD microspheres (Alg-RGD μS) were optionally precoated with 0.05% to 0.1% collagen/gelatin. Microspheres were cocultured with hMSCs at a ratio of 1:20 to 1:50 in low-adhesion centrifuge tubes for 30 to 60 min or in roller bottles at 10 rpm for 2 h. Unattached cells were removed by gentle washing to obtain hMSCs-Alg-RGD. Attachment efficiency was confirmed by DNA quantification or nuclear counting.

### Phenotypic characterization and molecular assays

Antibodies included CD90-FITC, CD105-APC, CD11b-PE, CD19-PE-Cy7, CD31-PerCP, and CD34-BV421. DAPI was used for live/dead cell staining. Gating strategy involved FSC/SSC → single cells → live cells → individual marker gating. FMO and isotype controls, as well as compensation matrices, were documented in the Supplementary Materials.

RNA was extracted using TRIzol, reverse-transcribed (random hexamers/oligo dT), and quantified with SYBR Green. The ΔΔCt method was used for relative quantification with GAPDH/B2M as internal references.

Cell viability was quantified using a CCK-8 (Beyotime Biotechnology, China). For 2D culture, hMSCs were seeded in 96-well plates at 1,000 cells per well. For 3D culture, Alg-RGD microbeads carrying an equivalent number of hMSCs (approximately 1,000 cells per well) were placed into 96-well plates. Cell viability was assessed on days 1, 2, 3, 4, and 5 after seeding using independent replicate wells for each time point. Briefly, culture medium was refreshed, and CCK-8 reagent was added at 10% (v/v). Plates were incubated at 37 °C, 5% CO_2_ for 1 to 2 h protected from light, and absorbance was measured at 450 nm using a microplate reader. Background absorbance from blank wells containing medium plus CCK-8 (and, for 3D groups, microbeads without cells) was subtracted.

Annexin V-FITC/PI or Annexin V-APC/7-AAD flow cytometry was performed to detect early/late-stage apoptosis.

Cells were incubated with 10 μM DCFH-DA at 37 °C for 30 min before imaging. Quantification was based on both background-subtracted mean fluorescence intensity and the percentage of positive cells.

Antibodies against α-SMA, vinculin, and YAP were used. F-actin was stained with Phalloidin-TRITC, and nuclei were stained with DAPI. Confocal microscopy parameters were kept consistent. The nuclear/cytoplasmic ratio of YAP was quantified by DAPI mask segmentation. Vinculin was quantified by measuring the combined area/number of focal adhesions and per-cell integrated intensity.

### HUVEC functional assays

EdU (10 μM, 2 h) was incorporated, and Click-iT chemistry was used for visualization. The percentage of positive cells was normalized to the DAPI count.

Cells were pretreated with serum-free medium for 6 h, and a scratch was made with a 200-μl pipette tip. The medium was then replaced with culture medium containing 1% FBS ± conditioned medium (CM). Images were taken at 12 and 24 h. Mitomycin C (2 μg/ml, 2 h) was used during key time points to control for proliferation interference. HUVECs were seeded at 2×10^4^ cells/well on prechilled Matrigel and imaged at 6 to 8 h. Angiogenesis Analyzer (ImageJ) was used to quantify total tube length, nodes, branches, and loops. In a Transwell (8 μm) assay, the upper chamber contained serum-free medium, while the lower chamber contained medium with 10% FBS or CM. After 24 h, cells were fixed, stained with crystal violet, and counted from images.

### Fibroblast (inflammatory/mechanical) assays

Fibroblasts were treated with 100 ng/ml LPS for 24 to 48 h. Groups included Control, LPS, LPS+2D-hMSC-CM, and LPS+hMSCs-Alg-RGD-CM. ROCK inhibitor (Y-27632, 10 μM), myosin II inhibitor (blebbistatin, 10 to 25 μM), or F-actin depolymerizing agent (latrunculin A, 100 nM) was added as needed to validate the involvement of mechanotransduction pathways.

### Mouse model and administration

This study and included experimental procedures were approved by the Institutional Animal Care and Use Committee of Shanghai Pulmonary Hospital, School of Medicine, Tongji University (approval number: K25-557). All animal housing and experiments were conducted in strict accordance with the institutional guidelines for the care and use of laboratory animals. ALI Model: ALI was induced by intratracheal instillation of LPS (5 mg/kg) under anesthesia. After 3 h, treatments were administered through an endotracheal tube, including hMSCs-Alg-RGD and GelMA@hMSCs-Alg-RGD (1 × 10^6^ hMSCs/mouse; 10 mg wet-weight microspheres).

Cells or microspheres were prelabeled with a near-infrared dye (DiR). Imaging was performed at 0, 6, 24, 48, and 72 h. A consistent region of interest (ROI) was used for the chest area. At the endpoint, lungs and other major organs were excised for ex vivo imaging. Bronchoalveolar lavage fluid (BALF) was collected with a fixed volume (3 × 0.5 ml of PBS). Total cell counts and differential counts were performed.

Flow cytometry was used to gate for neutrophil and macrophage populations. Lungs were fixed (at a constant pressure of 20 to 25 cm H_2_O), and paraffin sections were stained with hematoxylin and eosin (H&E) and Masson’s trichrome. Scoring was performed by a blinded pathologist. Lung homogenates or serum was used to measure interleukin-6 (IL-6), tumor necrosis factor-α (TNF-α), and interleukin-1β (IL-1β) (enzyme-linked immunosorbent assay), myeloperoxidase (MPO) activity, malondialdehyde (MDA), and superoxide dismutase (SOD) levels. The wet weight of the lung was measured, followed by drying at 60 °C to a constant dry weight to calculate the wet/dry weight ratio (W/D). Lung tissue immunofluorescence was performed with CD86 (M1) and CD206 (M2) antibodies. Quantification was based on the integrated intensity or percentage of positive area per field of view.

### Pulmonary function testing, arterial blood gas analysis, and hydroxyproline quantification

To evaluate short-term pulmonary mechanical function and oxygenation after LPS-induced ALI, respiratory mechanics testing and arterial blood gas analysis were performed at the acute time point after modeling in each group. Briefly, mice were anesthetized, tracheally intubated, and connected to a small-animal respiratory mechanics system (flexiVent FX system, SCIREQ, Canada). After stabilization under mechanical ventilation, respiratory system mechanics were acquired using the manufacturer’s standard protocols, including respiratory system elastance (Ers) and respiratory system resistance (Rrs). To obtain clinically relevant oxygenation endpoints, arterial blood was collected at the same time point as, or immediately adjacent to, pulmonary function testing for blood gas analysis. Blood gas parameters were measured using a portable blood gas analyzer (i-STAT, Abbott Laboratories, USA), and PaO_2_ was recorded.

To assess long-term tissue repair and fibrotic/collagen deposition trends, follow-up was extended to day 28 after modeling, and lung hydroxyproline content was quantified using a commercial assay kit (Hydroxyproline Assay Kit, Cat. No. A030-2-1).

### Statistical analysis

All quantitative data were analyzed using biological replicates as the statistical unit. Normality was assessed using the Shapiro–Wilk test, and homogeneity of variances was evaluated using Levene’s test (or the Brown–Forsythe test). For multigroup comparisons in which data were approximately normally distributed with homogeneous variances, one-way analysis of variance was performed followed by Tukey’s post hoc multiple-comparisons test. For data that did not meet normality and/or equal-variance assumptions, the Kruskal–Wallis test was applied followed by Dunn’s post hoc test, with multiple-comparisons correction performed as appropriate. Normally distributed data are presented as mean ± SD, whereas nonnormally distributed data are presented as median (interquartile range). A 2-sided *P* value < 0.05 was considered statistically significant. All analyses were performed via SPSS software.

## Results and Discussion

### Construction and characterization of hMSC 3D microenvironments

**Fig. 2.**
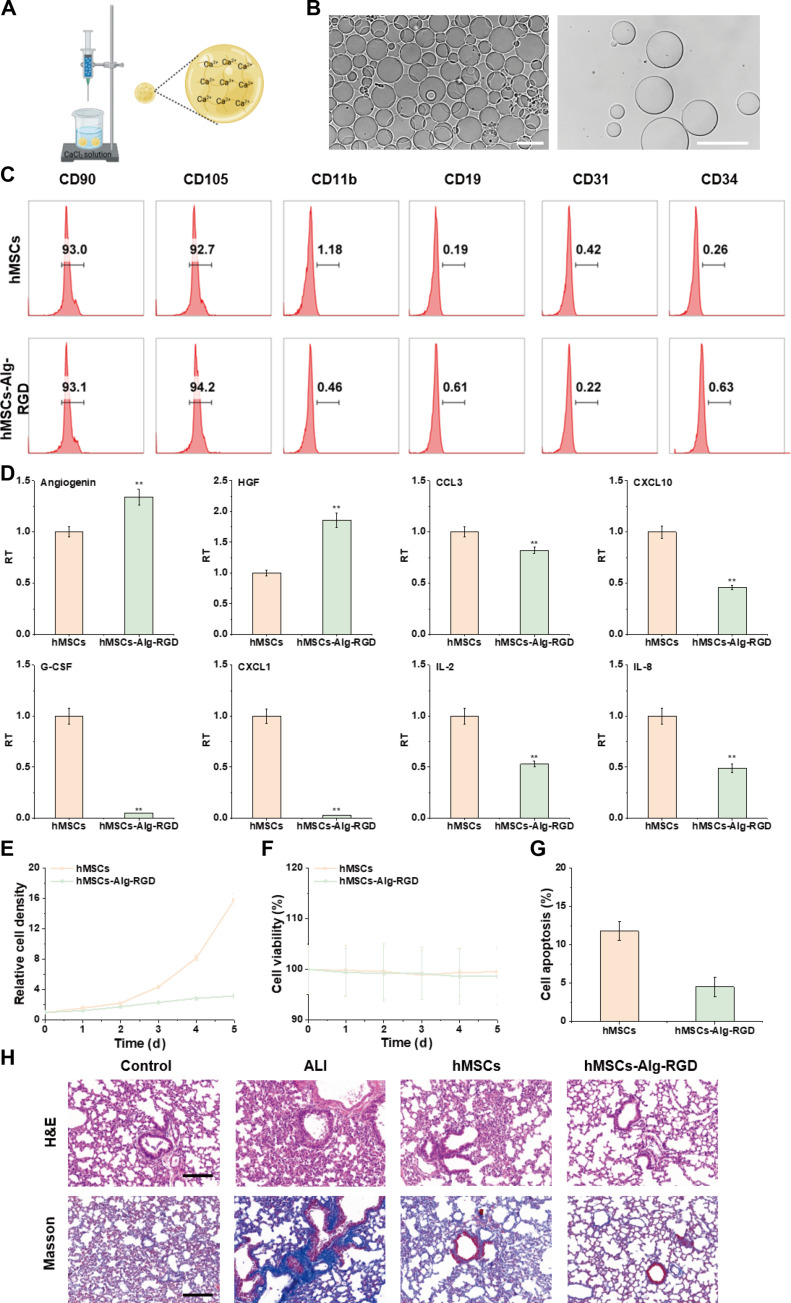
Preparation and characterization of hMSCs-Alg-RGD. (A) Schematic illustration of Alg-RGD preparation. (B) Representative optical microscopy image of Alg-RGD. Scale bar, 100 μm. (C) Flow cytometry analysis of marker expression in human mesenchymal stem cells (hMSCs) cultured under 2-dimensional (2D) conditions and hMSCs cultured with Alg-RGD in 3D, including CD90, CD105, CD11b, CD19, CD31, and CD34. (D) Polymerase chain reaction (PCR) comparison of marker expression between 2D-cultured hMSCs and 3D-cultured hMSCs-Alg-RGD (*N* = 5). (E) Cell density versus time curves for hMSCs cultured under 2D and 3D conditions. (F) Cell viability versus time curves for hMSCs cultured under 2D and 3D conditions. (G) Quantitative analysis of apoptosis rates in hMSCs after 2D and 3D culture (*N* = 5). (H) Representative hematoxylin and eosin (H&E) and Masson’s trichrome staining images of mouse lung tissues after different treatments. Scale bar, 100 μm. **P < 0.01.

We first focused on the construction and characterization of the hMSC 3D microenvironment. The fabrication workflow for alginate microbeads bearing RGD ligands (Alg-RGD μS) is shown in Fig. 2A: RGD peptides were grafted onto alginate carboxyls via EDC/NHS coupling, followed by emulsification/microfluidic processing to produce microbeads. Bright-field optical microscopy (Fig. 2B) clearly revealed favorable physical morphology, with a narrow size distribution, high sphericity, smooth surfaces, and uniform dispersion under hydrated conditions (Fig. S1). Such uniform microbeads are advantageous for homogeneous hMSC attachment and mass transport and also provide a basis for dense spreading over wet tissue surfaces. To evaluate how the 3D carrier alters intrinsic hMSC properties, we performed detailed cytometric analyses. Flow cytometry (Fig. 2C) showed that hMSCs maintained a canonical immunophenotype under both conventional 2-dimensional culture (2D-hMSCs) and after association with the 3D carrier (hMSCs-Alg-RGD): CD90 and CD105 were highly expressed, whereas hematopoietic/endothelial markers (CD11b, CD19, CD31, and CD34) were negative or low. This meets International Society for Cellular Therapy (ISCT) criteria for MSCs and strongly indicates that the 3D carrier did not change lineage identity. Subsequent qPCR analyses (Fig. 2D and Fig. S2) revealed deeper shifts: transcriptional levels of canonical MSC markers remained stable, whereas a subset of genes linked to paracrine and immunoregulatory function were up-regulated under 3D conditions. In contrast, transcripts associated with rapid proliferation decreased or remained unchanged, suggesting that the 3D carrier steers hMSCs toward a “high-paracrine/steady-state” rather than a “high-proliferation” phenotype—an orientation that is crucial for immunomodulatory and reparative function [21].

With respect to cell vitality and behavior, we longitudinally monitored hMSCs cultured in 2D versus 3D. The cell density–time curves (Fig. 2E) showed that 3D-cultured hMSCs rapidly completed attachment and cluster establishment during the early window (24 to 48 h) and then entered a gentle growth phase, exhibiting more stable colonization dynamics than 2D cultures. Viability–time curves (Fig. 2F) further supported this advantage: overall viability was higher and fluctuated less in the hMSCs-Alg-RGD group, indicating that the 3D microenvironment provides superior stress buffering and metabolic support. Quantitative apoptosis analyses (Fig. 2G and Fig. S3) corroborated this conclusion, showing lower apoptosis in the 3D group than in the 2D group and further demonstrating that Alg-RGD μS furnish a low-tension, low-stress niche for hMSCs [22]. Reduced apoptosis in the 3D hMSCs-Alg-RGD group likely reflects strengthened RGD–integrin anchorage that mitigates anoikis, together with a mechanically buffered 3D microenvironment that reduces stress concentration and supports pro-survival signaling. Notably, hMSCs-Alg-RGD also displayed robust antioxidative capacity; even after challenge with 200 mM H_2_O_2_, cell viability was effectively maintained. These in vitro characterizations provide a mechanistic basis for the therapeutic outcomes observed in vivo in a lung-injury model. Initial histological assessments (Fig. 2H and Fig. S5) showed that, relative to untreated or injury controls, the hMSCs-Alg-RGD treatment group exhibited more intact alveolar architecture and markedly reduced inflammatory infiltration on H&E staining, while Masson’s trichrome confirmed decreased collagen deposition in both area and density. Moreover, treated lungs displayed a trend toward M2 macrophage polarization (Fig. S6). These in vivo signatures of repair and antifibrotic potential align closely with the high viability, low apoptosis, and steady-state paracrine features observed for 3D cultures in vitro, collectively supporting the effectiveness of the material–cell composite strategy [23].

### Paracrine effects of hMSCs-Alg-RGD on endothelial function and macrophage polarization

**Fig. 3.**
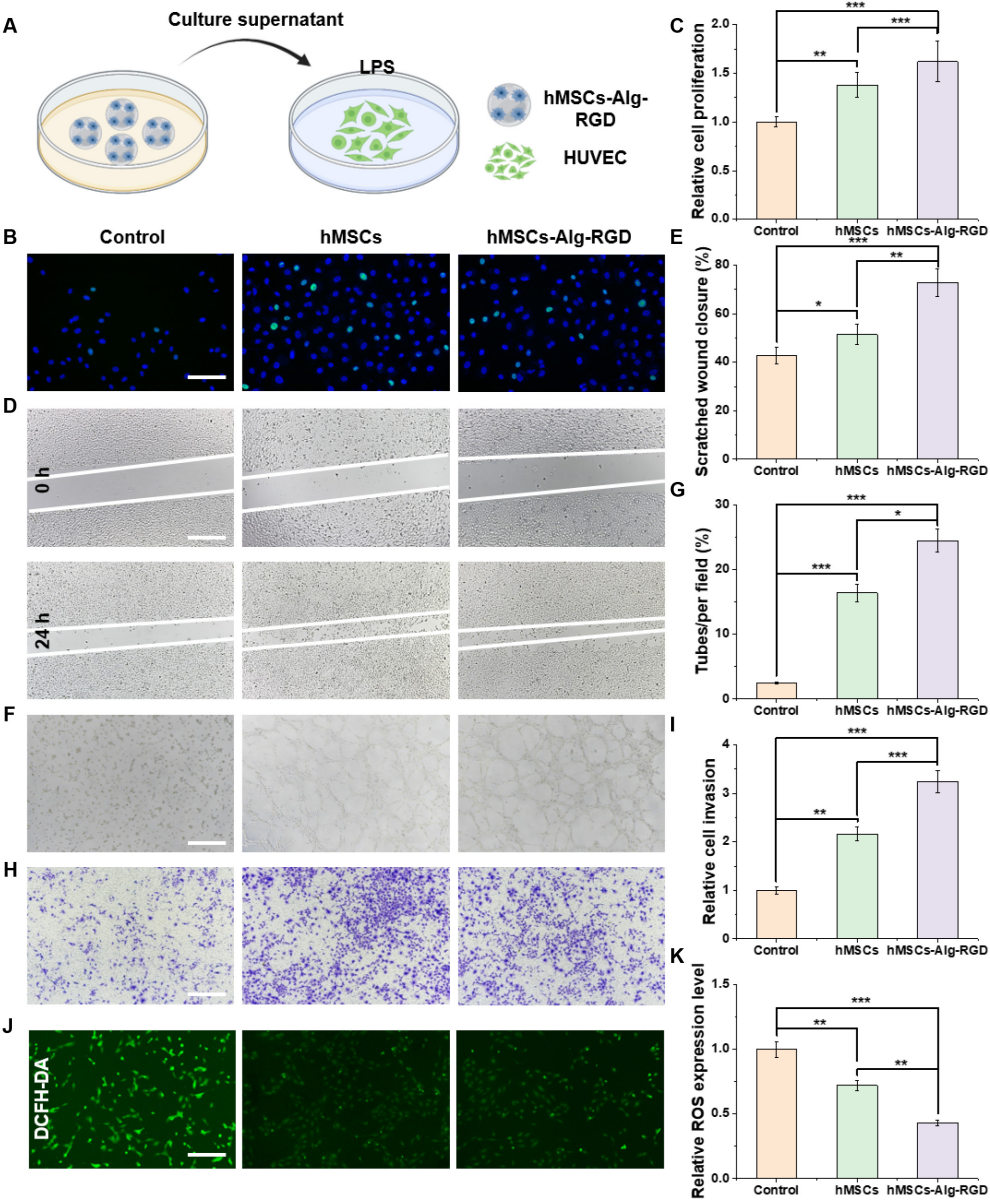
hMSCs-Alg-RGD. (A) Schematic illustration of the regulatory effects of hMSCs-Alg-RGD on human umbilical vein endothelial cells (HUVECs). (B) 5-Ethynyl-2′-deoxyuridine (EdU) staining analysis of cell proliferation in different groups (green: EdU; blue: 4′,6-diamidino-2-phenylindole [DAPI]). (C) Quantitative analysis of proliferation rates in each group (*N* = 3). (D) Scratch assay evaluating HUVEC migration capacity. (E) Quantitative analysis of cell migration (*N* = 3). (F) Tube formation assay assessing the pro-angiogenic potential of each group. (G) Quantitative analysis of tube formation efficiency (*N* = 3). (H) Transwell assay evaluating HUVEC invasion. (I) Quantitative analysis of cell invasion (*N* = 3). (J) Reactive oxygen species (ROS) staining analysis of intracellular oxidative stress in each group. (K) Quantitative analysis of ROS levels. Scale bar, 100 μm. *P < 0.05; **P < 0.01; ***P < 0.001.

To verify whether our hMSCs-Alg-RGD composites enhance the paracrine therapeutic effects of MSCs on vascular endothelial cells, we designed multidimensional in vitro functional assays, as schematically outlined in Fig. 3A. Conditioned media from hMSCs-Alg-RGD were used as the primary intervention and compared in parallel with untreated controls and 2D-hMSCs, allowing systematic evaluation of their influence on HUVECs in terms of proliferation, migration, angiogenesis, invasion, and oxidative stress. All representative images and quantifications correspond one-to-one with Fig. 3B to K, and significance levels match those annotated in the figure panels.

In the EdU incorporation assay (Fig. 3B and C), HUVECs exposed to hMSCs-Alg-RGD showed a markedly higher proportion of EdU-positive nuclei (green fluorescence) than controls (*P* < 0.001) and significantly higher than 2D-hMSCs (*P* < 0.01), normalized to total nuclei (blue, DAPI). This indicates that 3D-carrier-associated MSCs produce a stronger pro-growth paracrine profile. In wound-healing assays (Fig. 3D and E), the hMSCs-Alg-RGD group exhibited accelerated scratch closure. Representative images (Fig. 3D) showed greater advancement of wound edges within the same observation window, and quantification (Fig. 3E) revealed significantly higher closure ratios at 12 to 24 h compared to controls (*P* < 0.001) and 2D-hMSCs (*P* < 0.01). To exclude proliferative interference, mitomycin C was used to block cell division, and the enhanced migration advantage persisted, indicating that the effect stems primarily from strengthened migratory programs.

Matrigel-based angiogenesis assays (Fig. 3F and G) further confirmed this conclusion. The hMSCs-Alg-RGD group formed denser capillary-like networks with significantly increased numbers of branches, nodes, and total tube length. Quantitative analysis (Fig. 3G) demonstrated significantly elevated values for all parameters, with greater magnitude than 2D-hMSCs. These findings are consistent with the 3D paracrine signature observed on the MSC side, suggesting that vascular endothelial growth factor (VEGF), fibroblast growth factor (FGF), angiopoietin (ANGPT), and other angiogenic factors, along with EVs, synergistically drive the enhanced network formation capacity. In coated Transwell assays (Fig. 3H and I), the number of invading cells was significantly increased in the hMSCs-Alg-RGD group. Quantification (Fig. 3I) showed that the invasion index was significantly up-regulated versus both control and 2D-hMSCs (*P* < 0.001). Together with the migration and angiogenesis results, these findings demonstrate that 3D-carrier MSCs globally enhance endothelial motility and morphogenetic programs, which are essential for effective neovascular expansion into injured regions [24,25].

Finally, reactive oxygen species (ROS) staining (Fig. 3J and K) assessed oxidative stress. The hMSCs-Alg-RGD group displayed reduced intracellular ROS; representative images (Fig. 3J) showed visibly attenuated fluorescence intensity, and quantification (Fig. 3K) confirmed significantly reduced mean fluorescence intensity and percentage of ROS-positive cells. This aligns with the antioxidative phenotype of 3D-cultured MSCs and provides a mechanistic explanation for how reduced oxidative stress may couple to enhanced migration and tube formation capacity.

In addition to endothelial modulation, we also analyzed the impact of hMSCs-Alg-RGD on macrophage polarization. As shown in Fig. S7, both hMSCs and hMSCs-Alg-RGD reduced CD86 (M1 marker) and increased CD206 (M2 marker), with hMSCs-Alg-RGD showing a more pronounced effect. This indicates that hMSCs-Alg-RGD can effectively promote M2 polarization through paracrine signaling [17].

To further probe the underlying mechanisms, we examined whether the paracrine effects of hMSCs-Alg-RGD depend on the adhesion–contractility axis of hMSCs by introducing cytoskeletal inhibitors in the HUVEC assays (Fig. S8A). Treatment with the ROCK inhibitor Y-27632, the myosin II inhibitor blebbistatin, or the actin depolymerizer latrunculin A systematically tested the contribution of cytoskeletal mechanics. In EdU assays (Fig. S8B and F), the proliferative effect of hMSCs-Alg-RGD was significant but partially attenuated upon inhibitor treatment, suggesting that MSC adhesion–contractility contributes to paracrine-driven proliferation. Similarly, wound healing (Fig. S8C and G) revealed that migration enhancement by hMSCs-Alg-RGD was diminished in the presence of inhibitors, indicating that both soluble paracrine cues and mechanotransductive coupling shape migration outcomes. Transwell invasion (Fig. S8D and H) and Matrigel tube formation (Fig. S8E and I) yielded consistent findings: inhibitor addition significantly reduced invasion and angiogenesis, demonstrating that cytoskeletal contractility is indispensable for paracrine-mediated motility and morphogenesis. Collectively, these results indicate that the pro-angiogenic effects of hMSCs-Alg-RGD result from the combined action of paracrine factors and an endothelial-side permissive low-tension environment [26,27].

We further investigated whether cytoskeletal inhibitors affect hMSCs-Alg-RGD–mediated macrophage polarization (Fig. S9). When inhibitors were added in parallel to macrophage cultures, the M1→M2 transition normally induced by hMSCs-Alg-RGD was partially reversed. Representative immunofluorescence (Fig. S9A) showed that without inhibitors, hMSCs-Alg-RGD markedly reduced CD86 and increased CD206, whereas with inhibitors, the extent of CD86 reduction and CD206 elevation was blunted. Quantification (Fig. S9B and C) confirmed that CD86 levels were higher and CD206 levels were lower in inhibitor-treated groups compared to uninhibited hMSCs-Alg-RGD, though still improved relative to controls. This suggests that the primary paracrine effect persists, but the adhesion–contractility axis acts as a sensitizer of macrophage polarization, and when cytoskeletal tension is strongly inhibited, the M1→M2 shift is dampened.

### Suppression of myofibroblast differentiation via down-regulation of adhesion–tension signaling and mechanotransduction

**Fig. 4.**
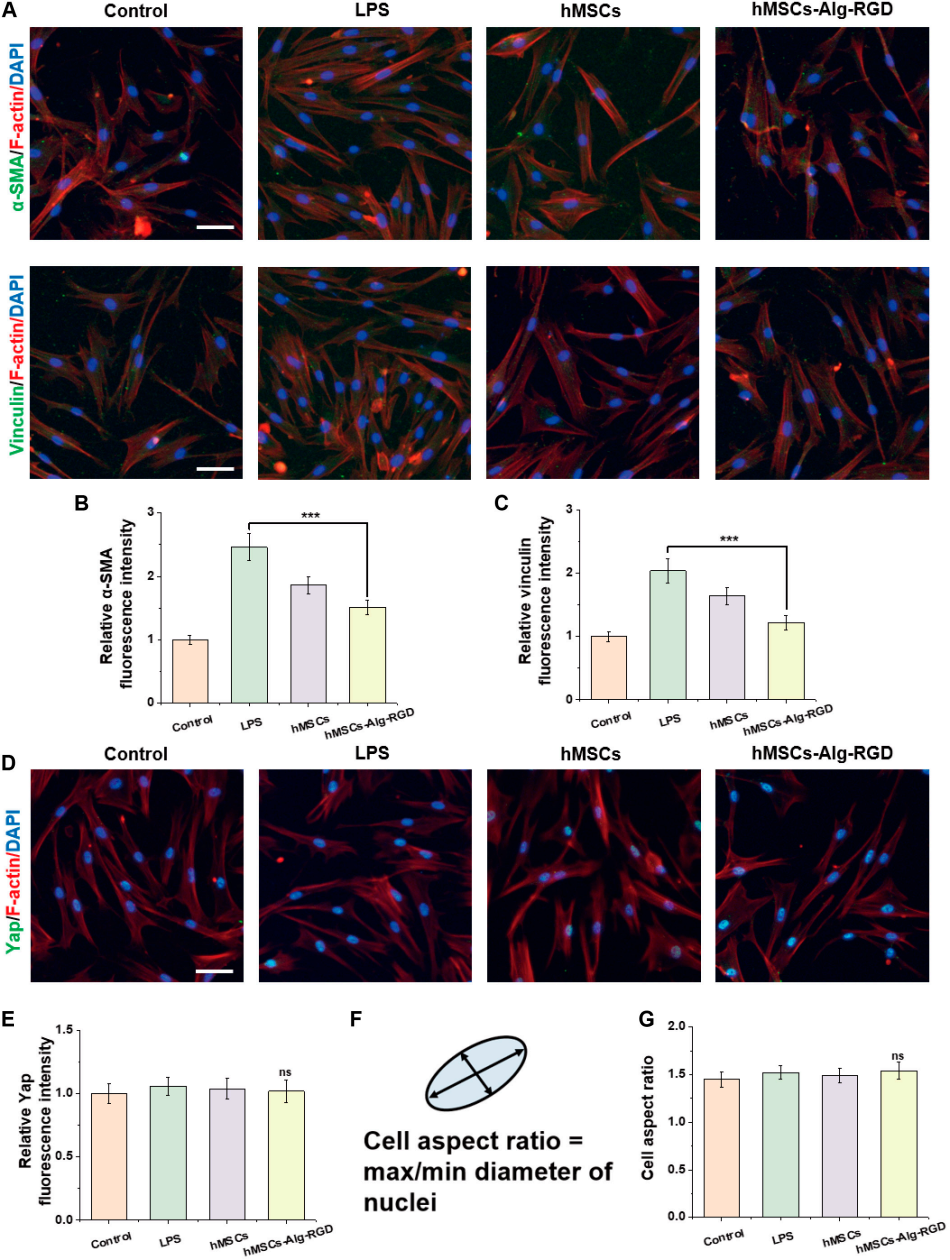
hMSCs-Alg-RGD. (A) Representative immunofluorescence images of fibroblasts after treatment with different materials, showing α-SMA and vinculin expression (green: target protein; red: F-actin; blue: DAPI). (B) Quantitative analysis of α-SMA immunofluorescence intensity across groups (*N* = 3). (C) Quantitative analysis of vinculin immunofluorescence intensity across groups (*N* = 3). (D) Representative immunofluorescence images of fibroblasts showing YAP expression after treatment (green: YAP; red: F-actin; blue: DAPI). (E) Quantitative analysis of YAP immunofluorescence intensity (*N* = 3). (F) Schematic illustration of cellular morphology features. (G) Quantitative analysis of cell aspect ratios in different groups (*N* = 3). Scale bar, 100 μm. ***P < 0.001.

To further elucidate the mechanisms by which our material–cell composite strategy suppresses fibrosis, we established a continuous readout system centered on “myofibroblast markers—adhesion/tension signaling—mechanotransduction—cell morphology”, as summarized in Fig. 4. Under injury/inflammatory stress (e.g., LPS stimulation), fibroblasts differentiate into myofibroblasts and display hallmark features such as α-SMA-positive stress fibers and mature vinculin-containing focal adhesions. Representative immunofluorescence images (Fig. 4A) show that in the LPS group, green α-SMA signals were aligned with red F-actin bundles, while vinculin appeared as large and bright puncta, reflecting activation of adhesion–tension pathways. In contrast, treatment with hMSCs-Alg-RGD markedly attenuated both signatures: α-SMA stress fibers became fragmented, and vinculin shifted from concentrated clusters to smaller and more dispersed puncta, indicating a global down-regulation of adhesion–contractile tension. Notably, when delivered in the sandwich format (i.e., microbeads encapsulated by top and bottom layers of wet-adhesive hydrogel), these “de-maturation” features were even more pronounced.

Quantitative analyses confirmed these observations. Compared to control and injury groups, α-SMA intensity was significantly reduced in treatment groups (Fig. 4B), with the hMSCs-Alg-RGD group showing a greater decrease than carrier-only controls. This highlights MSC paracrine factors as the primary driver of α-SMA suppression, amplified by the 3D carrier. Similarly, vinculin intensity was markedly decreased (Fig. 4C), mirroring the reduction in α-SMA, collectively supporting the conclusion that adhesion–contractile tension was systematically down-regulated [28].

We next assessed the nuclear–cytoplasmic distribution of the core mechanotransduction molecule YAP. In LPS-induced injury/inflammation controls, YAP signals appeared predominantly nuclear, consistent with activation under combined inflammatory and mechanical cues. After hMSCs-Alg-RGD treatment, YAP staining showed a more diffuse peri-nuclear/cytoplasmic pattern in representative images (Fig. 4D), which was directionally consistent with the de-maturation of stress fibers and focal adhesions. Quantification (Fig. 4E) indicated a downward trend in YAP nuclear localization; however, this difference did not reach statistical significance (ns) under the current sample size and analysis pipeline. Accordingly, we interpret YAP as a trend-level readout rather than a definitive mechanistic endpoint, while the significant reductions in α-SMA and vinculin provide the primary evidence for attenuated adhesion–tension signaling and myofibroblast activation [29].

Finally, to relate biomechanical readouts to cell morphology, we conducted morphometric analyses (Fig. 4F). Fibroblasts typically adopt an elongated, polarized morphology under high-tension/stiff environments. Compared with control and injury groups, treated cells tended to show reduced elongation (Fig. 4G and Fig. S10), suggesting a shift toward a less polarized morphology. Together, the significant decreases in α-SMA and vinculin indicate suppressed fibroblast activation and adhesion maturation, whereas YAP nuclear localization and elongation provide directionally consistent but nonsignificant trends that warrant further validation [30].

### Design and characterization of GelMA@hMSCs-Alg-RGD for prolonged pulmonary retention

Although our previous experiments demonstrated the broad therapeutic potential of hMSCs-Alg-RGD for ALI, effective treatment outcomes remained limited because transplanted cells readily migrate away from the lesion site. To address this limitation, we developed a hydrogel carrier, GelMA@hMSCs-Alg-RGD, designed to achieve long-term retention at pulmonary injury sites. This composite not only prolongs local residence of hMSCs-Alg-RGD but also ensures favorable biosafety.

**Fig. 5.**
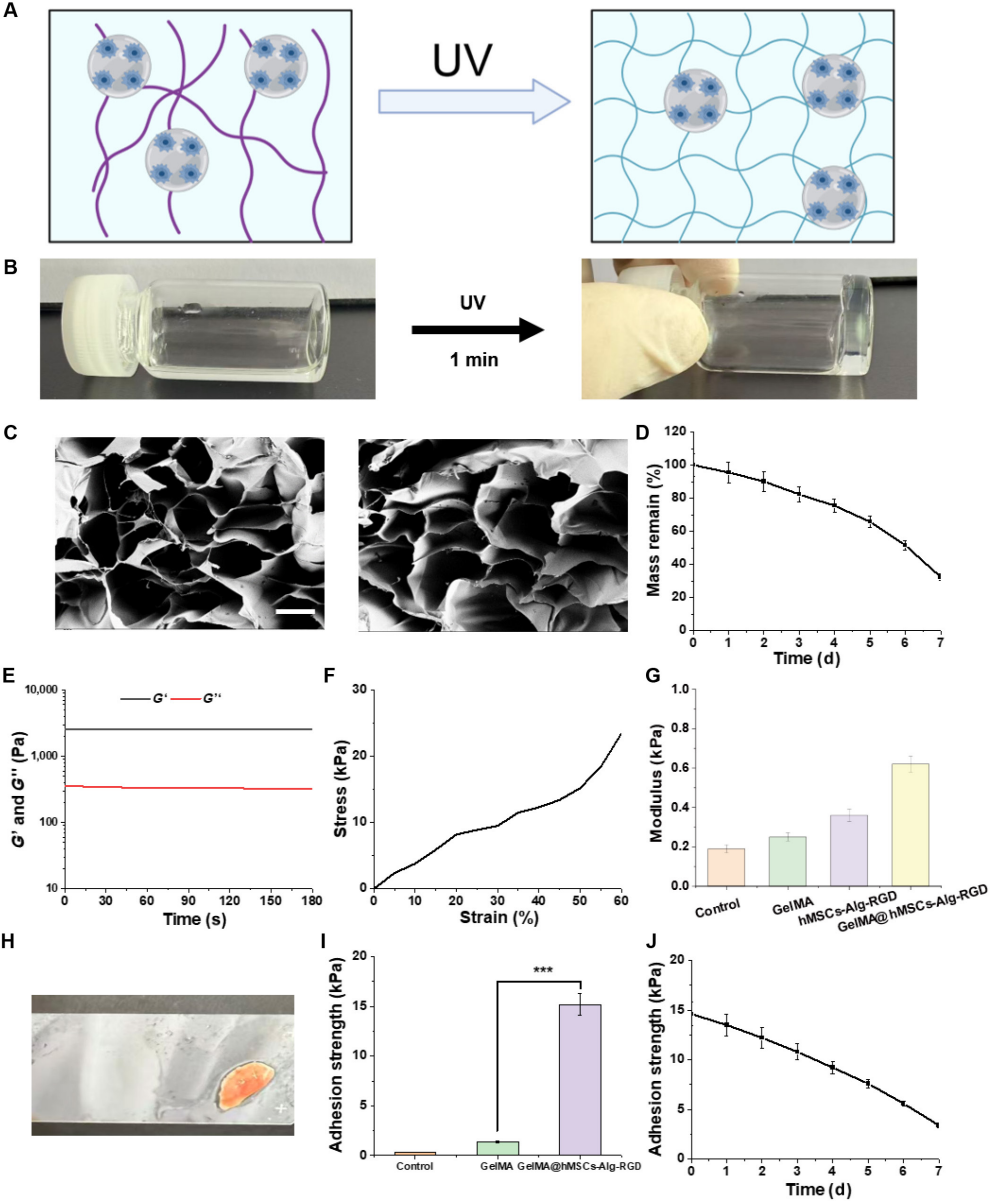
Preparation and characterization of GelMA@hMSCs-Alg-RGD. (A) Schematic illustration of the preparation of GelMA@hMSCs-Alg-RGD composite hydrogel. (B) Rapid photo-crosslinking of GelMA@hMSCs-Alg-RGD under ultraviolet (UV) irradiation. (C) Representative scanning electron microscopy (SEM) image of GelMA@hMSCs-Alg-RGD showing the integrated microstructure with embedded Alg-RGD microspheres. Scale bar, 100 μm. (D) Degradation profile of GelMA@hMSCs-Alg-RGD in enzymatic solution. (E) Rheological curves of GelMA@hMSCs-Alg-RGD. (F) Stress–strain curves of GelMA@hMSCs-Alg-RGD under quasi-static loading. (G) Quantitative modulus analysis of different groups (*N* = 3). (H) Demonstration of the wet-adhesion property of GelMA@hMSCs-Alg-RGD, stably attaching moist mouse lung tissue onto a glass slide. (I) Quantitative analysis of adhesion strength among different groups (*N* = 5). (J) Time-dependent adhesion strength curves of GelMA@hMSCs-Alg-RGD. Scale bar, 100 μm. ***P < 0.001.

We first comprehensively characterized the key physicochemical and mechanical properties of GelMA@hMSCs-Alg-RGD. The construction workflow is illustrated in Fig. 5A, in which Gel-DA layers are sequentially applied above and below hMSCs-Alg-RGD microbeads, forming a “sandwich” architecture that enables in situ fixation and localized delivery on moist tissue surfaces. The composite demonstrated rapid responsiveness and robust shaping capability: under ultraviolet (UV) irradiation, the upper and lower Gel-DA layers transitioned from sol to gel within seconds to minutes (Fig. 5B). This rapid gelation allows fast adhesion and conformation on wet surfaces, meeting the temporal demands of endoscopic or minimally invasive procedures [31].

Representative scanning electron microscopy (SEM) images (Fig. 5C) showed a continuous and dense gel matrix with Alg-RGD microbeads tightly embedded, without visible interfacial delamination or voids. This architecture facilitates mechanical interlocking between layers while maintaining mass transport channels for oxygen, nutrient exchange, and secretion diffusion—providing a structural foundation for sustained cell viability and paracrine function [17].

The degradation profile of the composite was carefully evaluated. In enzymatic environments, GelMA@hMSCs-Alg-RGD exhibited a gradual and controlled degradation curve, maintaining structural integrity throughout the therapeutic window while avoiding risks associated with long-term residue (Fig. 5D). Its half-life and residual fraction aligned well with the in vivo retention timeframes observed in the main study.

Mechanical analyses further highlighted the material’s compatibility with pulmonary tissue. Rheological measurements (Fig. 5E) showed that after gelation, the storage modulus (*G*′) rapidly increased and stabilized, remaining greater than the loss modulus (*G*″), indicative of a stable crosslinked network. Frequency sweeps revealed a medium-to-low *G*′ with nonzero *G*″, reflecting viscoelastic dissipation and stress relaxation capacity. These properties are crucial for dispersing peak stresses during respiratory motion and reducing tension on both cells and lung tissue. Quasi-static stress–strain curves (Fig. 5F) confirmed mechanical compliance: the material exhibited low initial modulus followed by modest reinforcement, enabling good conformability to the soft, deformable lung surface while preserving extensibility and integrity within physiological strain ranges. Quantitative modulus analysis (Fig. 5G) placed GelMA@hMSCs-Alg-RGD within the softness range of healthy lung tissue and significantly lower than stiffer controls, directly supporting our design principle of a “low-tension, mechanically friendly” carrier that helps suppress YAP/adhesion–contractility pathway overactivation.

To provide physiological context, reported mechanical properties of human lung parenchyma are generally in the soft-tissue (kPa-scale) regime, with substantially increased stiffness in fibrotic remodeling. We therefore interpret our rheology and relaxation data as confirming that the hydrogel remains mechanically compliant and dissipative under respiration-relevant deformation. Importantly, the hydrogel in this study was not optimized for precise modulus matching; instead, wet-adhesion strength and durability were used as the primary criteria to enable prolonged local retention on the dynamic lung surface. As demonstrated in Fig. 5H, GelMA@hMSCs-Alg-RGD achieved strong adhesion between moist mouse lung tissue and glass substrates, allowing tissue slices to be lifted and inverted without detachment. This capability arises from catechol-mediated covalent and noncovalent interactions coupled with surface roughness–enhanced interfacial energy, validating robust underwater adhesion even in complex biofluids such as blood or mucus. Quantitative adhesion strength tests (Fig. 5I) showed significantly higher pull-off and shear strengths for GelMA@hMSCs-Alg-RGD compared to controls, with lower variance, indicating reproducible and predictable fixation performance. The time course adhesion data (Fig. 5J) shows that the hydrogel can still maintain the adhesion strength of 4.3 kPa after 7 days, which defines a practical intraoperative window for repositioning or closing and alignment with the prolonged retention track observed in the main study.

In summary, through multidimensional characterization spanning gelation kinetics, microstructure, degradability, viscoelasticity, and underwater adhesion, we established that GelMA@hMSCs-Alg-RGD possesses essential attributes of rapid in situ gelation, mechanical softness with stress relaxation, stable wet adhesion, and controlled degradability. These material properties provide the mechanistic foundation for the extended pulmonary retention, attenuation of inflammatory/mechanical signaling, and histological improvements observed in vivo.

### Prolonged in vivo retention of GelMA@hMSCs-Alg-RGD in the pulmonary microenvironment

**Fig. 6.**
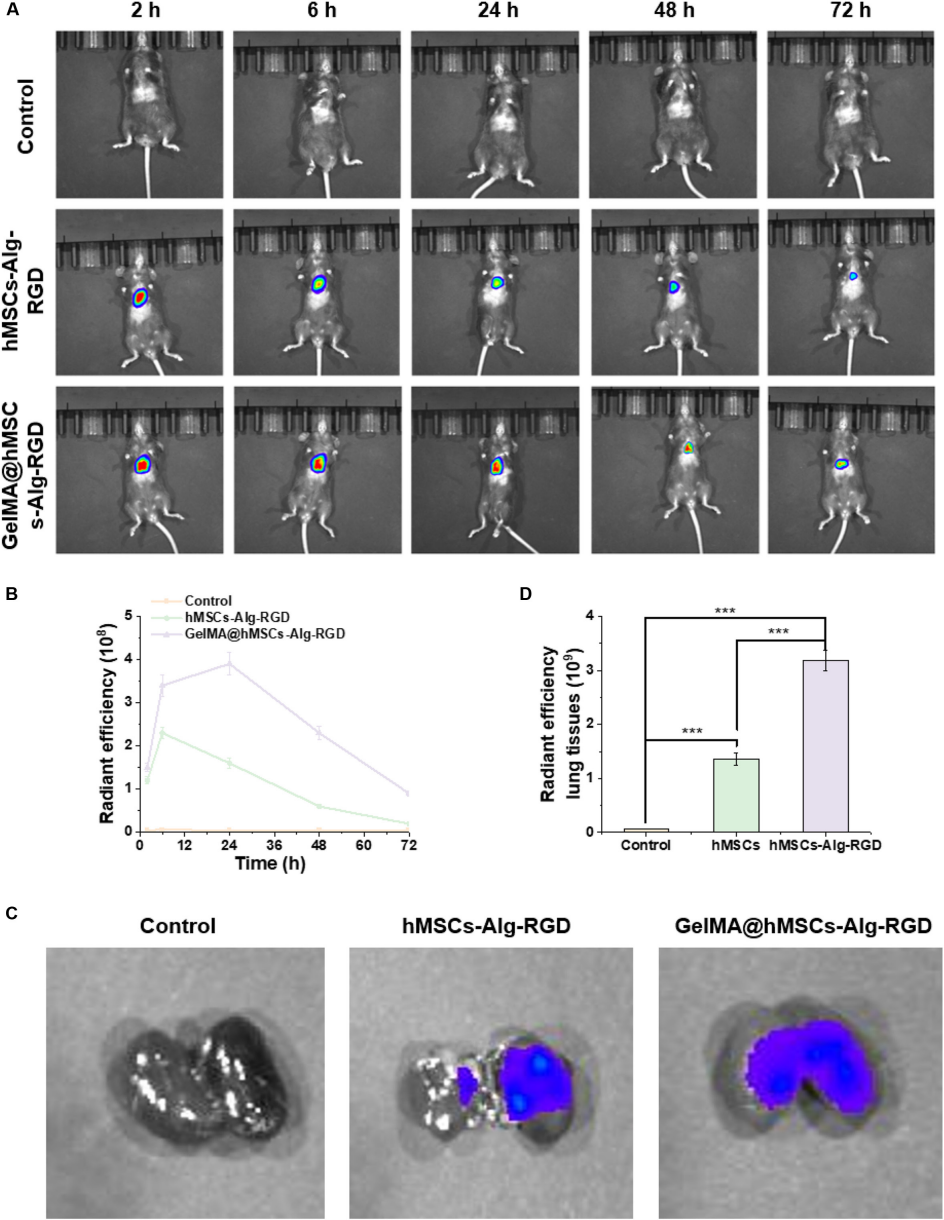
In vivo biodistribution and pulmonary retention of different materials. (A) Representative in vivo fluorescence imaging of mice after administration of different materials. (B) Time-dependent fluorescence intensity curves of mice across groups (*N* = 3). (C) Representative ex vivo fluorescence images of isolated lung tissues. (D) Quantitative analysis of lung tissue fluorescence intensity in each group (*N* = 3). ***P < 0.001.

To evaluate the retention capacity of the composite in the dynamic lung environment, we employed small-animal in vivo imaging to track the biodistribution and persistence of different treatment groups. Representative bioluminescence/fluorescence images are shown in Fig. 6A. Under identical acquisition parameters, only background-level systemic signals were observed in control or carrier-only groups. In contrast, mice receiving hMSCs-Alg-RGD microbeads exhibited distinct focal fluorescence in the thoracic region, but the signal gradually decayed over time. Notably, the “sandwich delivery” group GelMA@hMSCs-Alg-RGD displayed fluorescence that was more concentrated, uniform, and decayed more slowly. This finding indicates that the wet-adhesive Gel-DA layers firmly anchored the microbead–cell complexes on lesion surfaces, effectively resisting migration and clearance induced by respiratory motion and body fluid flushing. Furthermore, across multiple animals, the sandwich group showed no abnormal signal aggregation in off-target sites such as the liver, spleen, or peritoneal cavity, suggesting favorable local confinement and controlled biodistribution.

Quantitative analysis of predefined thoracic ROIs further confirmed the superiority of sandwich delivery (Fig. 6B). Kinetic curves recorded over 0 to 72 h revealed that signals in control and carrier-only groups rapidly decayed to baseline. The hMSCs-Alg-RGD group followed an exponential decay with a relatively short half-life. In contrast, the sandwich group curve was consistently elevated with a shallower slope, indicating markedly prolonged in situ retention. At all time points, thoracic fluorescence in the sandwich group exceeded that in the hMSCs-Alg-RGD group, with the most pronounced differences occurring at mid-to-late stages (24 to 48 h), reflecting the dual benefits of initial anchoring and sustained resistance to washout.

To corroborate local material retention at the experimental endpoint, we performed ex vivo imaging of major organs. As shown in Fig. 6C, patchy fluorescence was visible on the lung surfaces of the hMSCs-Alg-RGD group, but the boundaries appeared fragmented. In contrast, lungs from the sandwich delivery group exhibited continuous, well-defined fluorescent “patches,” spatially consistent with the thoracic hotspots seen in vivo. This observation demonstrates that the Gel-DA thin layers effectively maintained adhesion and conformation on moist tissue throughout the experimental period. Importantly, no abnormally high signals were detected in other organs (liver, spleen, and kidney), confirming minimal ectopic accumulation or distal migration. Quantitative ROI analysis of excised lung tissues (Fig. 6D) revealed that normalized fluorescence intensity was significantly higher in the sandwich group compared to hMSCs-Alg-RGD and controls.

Taken together with the in vivo kinetic data, these results establish that the sandwich layers not only increased the initial local dose but also extended the duration of effective residence, ultimately translating into higher lung accumulation at endpoint ex vivo readouts. This outcome aligns with the histological and functional improvements previously observed—such as reduced inflammation and decreased collagen deposition—providing strong support for the causal chain of “retention → local exposure → therapeutic efficacy”.

### Therapeutic efficacy of GelMA@hMSCs-Alg-RGD in a murine lung injury model

Before in vivo studies, we systematically evaluated the biosafety of GelMA@hMSCs-Alg-RGD. As shown in Figs. S11 to S13, no significant histopathological differences were observed across major organs, and serum biochemical parameters remained within normal ranges, indicating the absence of overt toxicity. In addition, longitudinal body-weight monitoring during the acute observation window revealed no abrupt posttreatment weight loss compared with the control groups (Fig. S14), further supporting good tolerability and minimal systemic stress induced by the formulations.

To assess the therapeutic potential of our material–cell composite, we established a murine lung injury model and applied the GelMA@hMSCs-Alg-RGD sandwich construct locally to the lung surface using laryngoscope/tracheal-guided intratracheal delivery. This strategy highlights the engineering advantages of local retention and mechanical compatibility. A matrix of multi-endpoint assessments was subsequently performed, encompassing lung water content, histological analyses, and inflammatory and oxidative stress biomarkers.

**Fig. 7.**
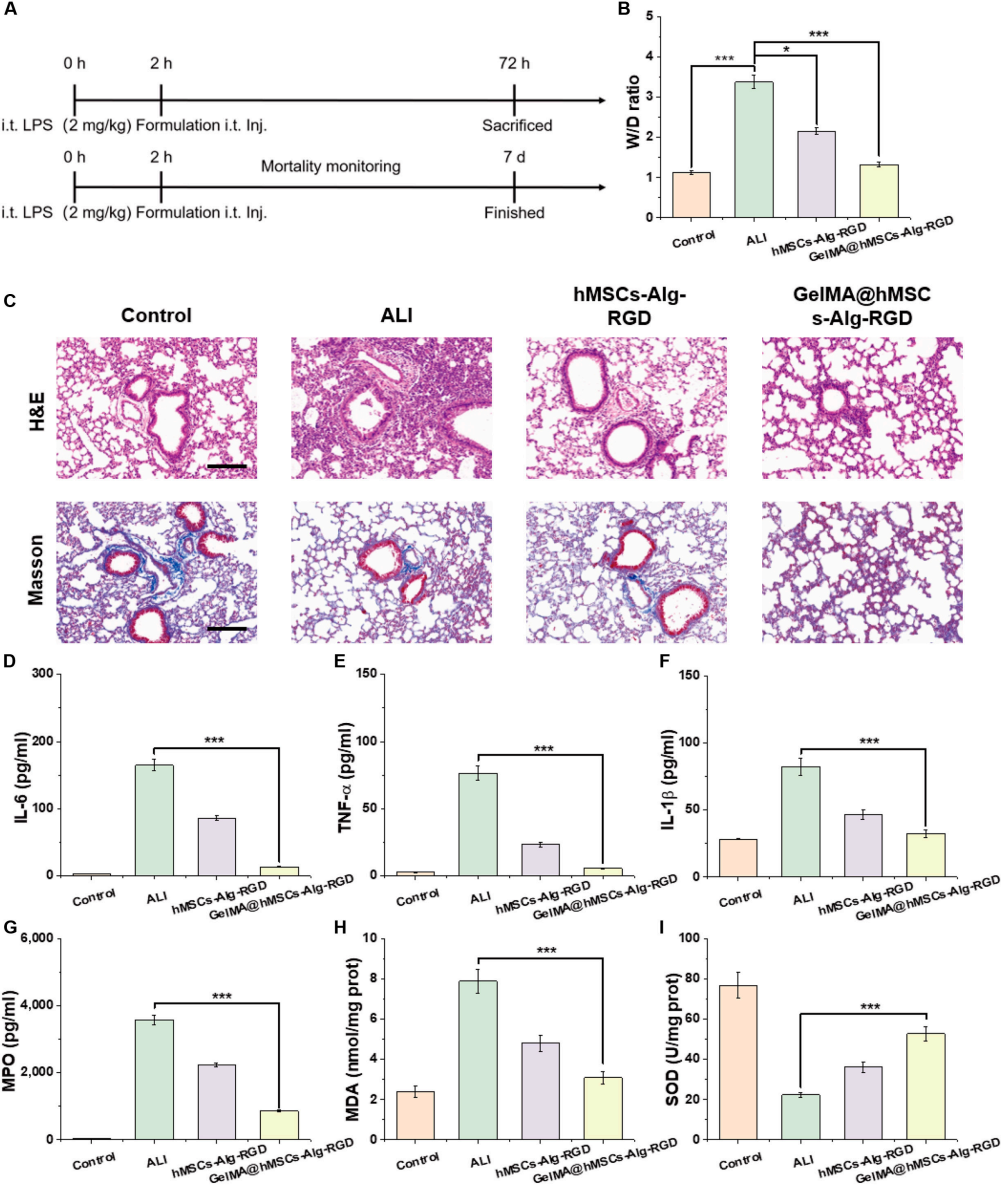
Therapeutic efficacy of GelMA@hMSCs-Alg-RGD in a murine lung injury model. (A) Schematic illustration of the treatment procedure with GelMA@hMSCs-Alg-RGD for acute lung injury. (B) Lung wet/dry weight ratio (W/D) of mice after treatment across groups (*N* = 5). (C) Representative H&E and Masson’s trichrome staining images of lung tissues after treatment. (D to I) Quantitative analysis of inflammatory and oxidative stress markers, including (D) interleukin-6 (IL-6), (E) tumor necrosis factor-α (TNF-α), (F) interleukin-1β) (IL-1β), (G) myeloperoxidase (MPO), (H) malondialdehyde (MDA), and (I) superoxide dismutase (SOD) (*N* = 5). Scale bar, 100 μm. *P < 0.05; ***P < 0.001.

Measurement of lung W/D (Fig. 7B) revealed a general reduction across all treatment groups, with the greatest and most significant decrease observed in the GelMA@hMSCs-Alg-RGD group. These findings demonstrate that sandwich delivery provides the most effective protection against interstitial and alveolar edema, thereby substantiating the causal chain of “in situ retention ↑ → effective local exposure ↑ → vascular leakage/exudation ↓”.

Histological analyses further corroborated these therapeutic benefits (Fig. 7C and Fig. S15A and B). In H&E-stained sections, the injury control group exhibited thickened alveolar septa, hemorrhage, and diffuse inflammatory cell infiltration. In contrast, lungs from the GelMA@hMSCs-Alg-RGD group displayed relatively preserved alveolar architecture, thinner septa, and markedly reduced inflammatory infiltrates. Masson’s trichrome staining confirmed the lowest collagen deposition area and density in the sandwich-treated group, consistent with the H&E results and suggesting concomitant mitigation of both inflammation and fibrosis.

Quantitative analyses of inflammatory and oxidative stress markers reinforced these conclusions. Levels of IL-6, TNF-α, and IL-1β were significantly reduced in treatment groups compared with injury controls (Fig. 7D to F), with the greatest reductions observed in GelMA@hMSCs-Alg-RGD, which also retained statistical superiority over hMSCs-Alg-RGD alone. These results align with the in vitro findings of down-regulated adhesion–tension and YAP signaling, confirming that synergistic paracrine and immunomodulatory functions of the material–cell construct were effectively reproduced in vivo. As a surrogate for neutrophil infiltration and activation, MPO activity was also significantly decreased in the GelMA@hMSCs-Alg-RGD group (Fig. 7G), consistent with the reduction in inflammatory cells observed histologically. Similarly, indices of oxidative stress were most favorably modulated by GelMA@hMSCs-Alg-RGD: MDA, a lipid peroxidation product, was lowest (Fig. 6H), while SOD activity was most elevated (Fig. 6I). Importantly, survival rates were markedly improved in GelMA@hMSCs-Alg-RGD-treated mice (Fig. S15C).

Collectively, these cross-level, consistent datasets demonstrate that the sandwich delivery strategy not only alleviates inflammation but also attenuates oxidative stress and lipid peroxidation, thereby achieving comprehensive protection in ALI.

### Immunomodulatory effects of GelMA@hMSCs-Alg-RGD on neutrophil infiltration and macrophage polarization

**Fig. 8.**
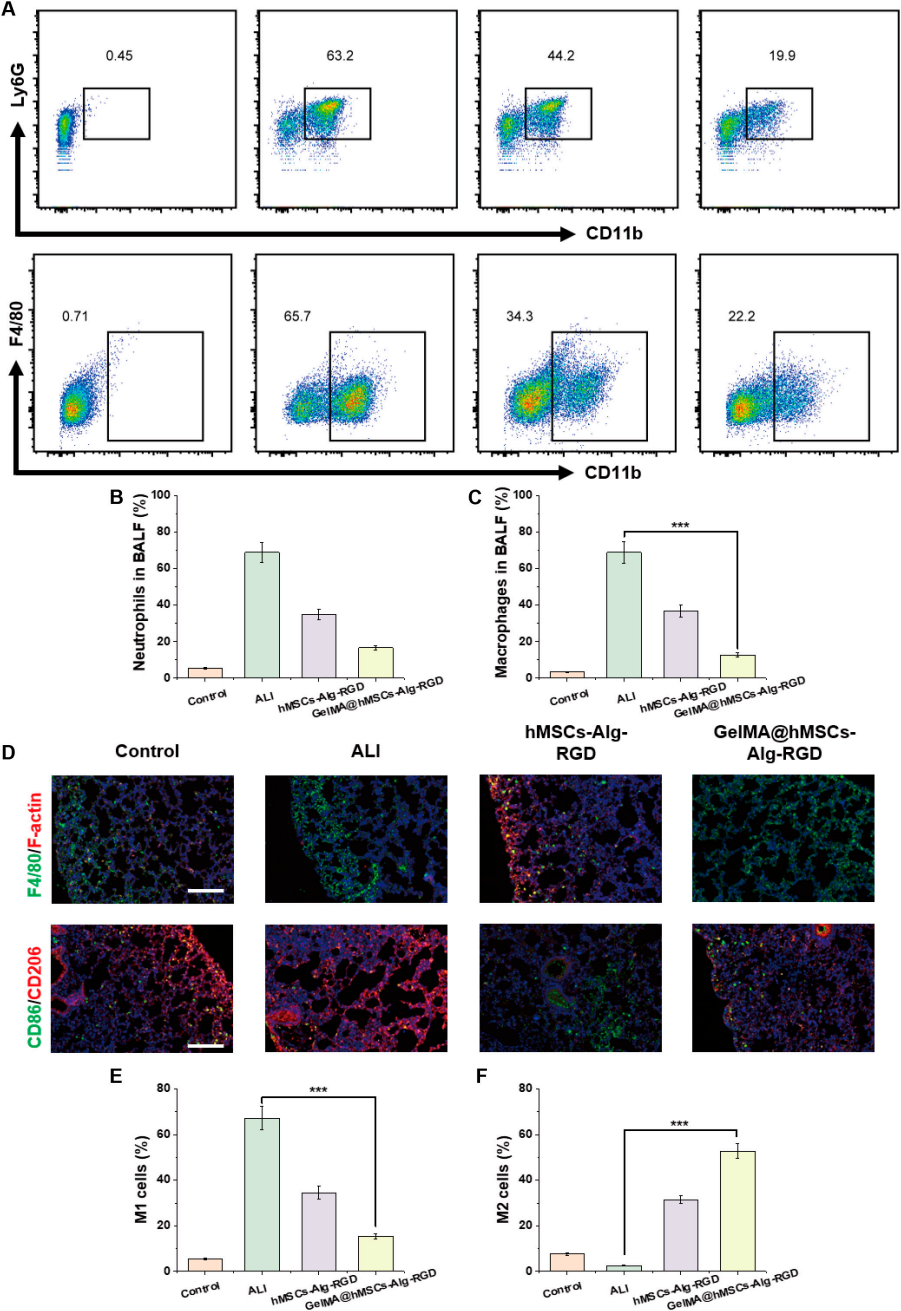
Immunomodulatory effects of GelMA@hMSCs-Alg-RGD in vivo. (A) Representative flow cytometry dot plots of neutrophils and macrophages in bronchoalveolar lavage fluid (BALF) from different groups. (B) Quantitative analysis of neutrophil counts (*N* = 5). (C) Quantitative analysis of macrophage counts (*N* = 5). (D) Representative immunofluorescence images of lung tissues showing macrophage polarization markers CD86 and CD206. (E) Quantitative analysis of M1 marker CD86 expression (*N* = 5). (F) Quantitative analysis of M2 marker CD206 expression (*N* = 5).

To further elucidate the immunomodulatory mechanisms of our composite, we analyzed BALF and lung tissue. Flow cytometry (gating shown in Fig. 8A) demonstrated that, compared with the ALI injury group, both neutrophils (CD45^+^Ly6G^+^CD11b^+^) and macrophages (CD45^+^Ly6G^−^CD64^+^F4/80^+^) decreased after treatment, with the sandwich construct showing the largest reduction. In contrast, treatment groups—particularly GelMA@hMSCs-Alg-RGD—exhibited a significant reduction in neutrophils accompanied by restoration and expansion of the macrophage population. These findings indicate that our strategy attenuates overall inflammatory infiltration while reestablishing macrophage homeostasis in a reparative direction.

Quantitative analyses confirmed these results. Neutrophil numbers (Fig. 8B) were significantly reduced in the hMSCs-Alg-RGD group and declined even further in the GelMA@hMSCs-Alg-RGD group, consistent with the observed reduction in MPO activity reported earlier. Meanwhile, macrophage numbers (Fig. 8C) increased after treatment, with the most pronounced recovery in the sandwich delivery group. This combination of “neutrophils ↓ + macrophages ↑” suggests that the material–cell composite not only suppresses acute granulocytic inflammatory cascades but also facilitates the restoration of “scavenger/repair-oriented” innate immune cells.

To further delineate macrophage phenotypes, we performed dual immunofluorescence staining of lung sections for CD86 (M1 marker) and CD206 (M2 marker) (Fig. 8D). In injury controls, representative images showed dominant CD86 signals, extensive inflammatory infiltration, and thickened septa. In the hMSCs-Alg-RGD group, CD86 signals weakened while CD206 signals increased. Most notably, in the GelMA@hMSCs-Alg-RGD group, CD86 was further reduced and CD206 strongly enhanced, indicating a robust shift toward M2 (pro-resolving/pro-repair) polarization. Quantitative results (Fig. 8E and F) supported these observations: both treatments significantly decreased CD86 expression, but the sandwich group exhibited the greatest reduction (Fig. 8E). Conversely, CD206 expression was significantly up-regulated in both treatments, with the highest increase again observed in the sandwich group (Fig. 8F and Fig. S16).

Taken together, these results demonstrate that GelMA@hMSCs-Alg-RGD achieved the most pronounced M1→M2 transition, underscoring how enhanced in situ retention × amplified 3D MSC paracrine activity synergize to achieve comprehensive immune microenvironment remodeling.

## Conclusion

In summary, we developed and validated GelMA@hMSCs-Alg-RGD as a next-generation MSC delivery strategy for ALI. By combining RGD-functionalized alginate microbeads that enhance MSC viability and paracrine activity with an adhesive, stress-relaxing Gel-DA hydrogel that ensures in situ retention, this composite simultaneously addresses the 2 major barriers to MSC therapy: poor localization and insufficient immunomodulation. Mechanistically, it establishes a continuous causal chain from prolonged pulmonary residence and enhanced local exposure to suppression of adhesion–tension signaling, fibroblast activation, and inflammatory cytokines, culminating in improved tissue structure, reduced fibrosis, and increased survival. Beyond ALI, this modular design principle provides a versatile platform for regenerative medicine where spatiotemporal control of MSC paracrine activity is essential. Importantly, this platform also offers a practical pathway toward clinical translation from a manufacturing perspective. Both alginate and gelatin/gelatin derivatives are well-established, widely used biomaterials with scalable supply chains, and the key modification/curing steps have well-defined process windows that support standardized standard operating procedures (SOPs). Moreover, Alg-RGD microbeads can be produced using scalable fabrication modalities, with size distribution and key quality attributes readily monitored through online/offline measurements to ensure batch-to-batch consistency.

## Data Availability

Research data supporting this publication are available upon request.
