# Supplementary material for: A Mechanotransduction-Aware Strategy for Enhancing MSC Potency via 3D Culture and Localized Delivery
Source: Cyborg Bionic Syst. 2026 Mar 24;7:0552. doi: 10.34133/cbsystems.0552 (PMC13009532; doi:10.34133/cbsystems.0552)
Supplement: Supplementary 1 — Figs. S1 to S16 [file cbsystems.0552.f1.zip › SUPPLEMENTARY MATERIALS.docx]

SUPPLEMENTARY MATERIALS


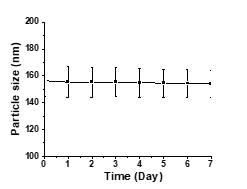


Figure S1. Time-dependent particle size changes of Alg-RGD microspheres. The hydrodynamic diameter of Alg-RGD microspheres was continuously monitored over time, revealing stable size maintenance without significant aggregation or swelling, thus confirming their colloidal stability in aqueous environments.


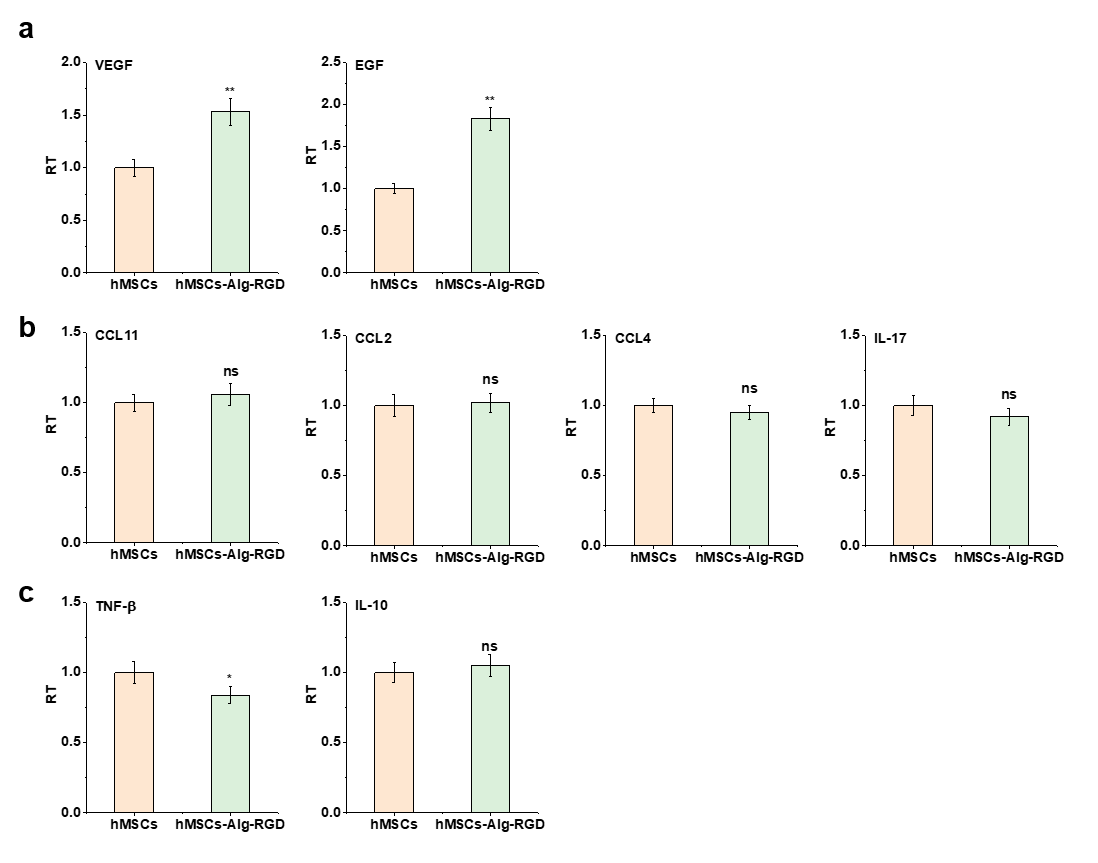


Figure S2. PCR comparison of lineage-specific marker expression between 2D-cultured hMSCs and 3D-cultured hMSCs-Alg-RGD. The results show that MSC canonical markers were preserved in both groups, while 3D culture promoted the upregulation of genes associated with paracrine and immunomodulatory functions.


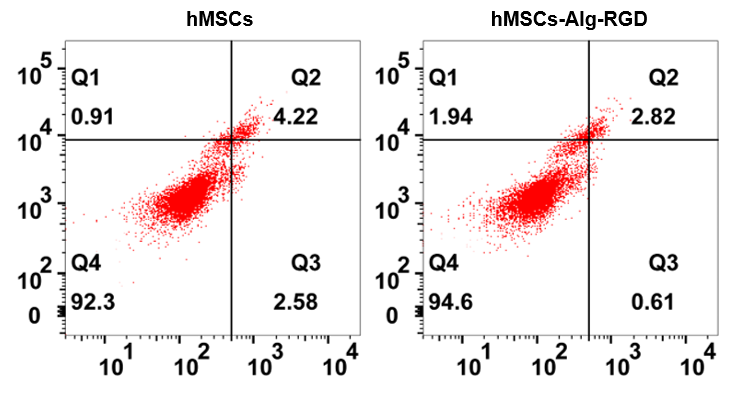


Figure S3. Representative flow cytometry images showing apoptosis of hMSCs cultured in 2D versus 3D (hMSCs-Alg-RGD). Quantitative analysis demonstrated a significantly reduced apoptotic fraction in the 3D culture condition, indicating the protective effect of the Alg-RGD microenvironment.


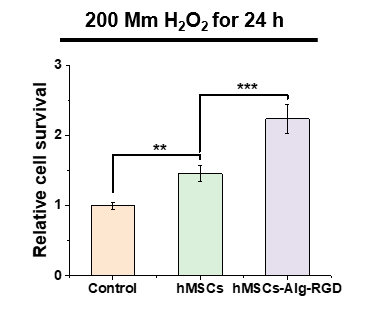


Figure S4. Antioxidant capacity of hMSCs-Alg-RGD. The 3D encapsulation markedly enhanced the cellular resistance against oxidative stress induced by H₂O₂, maintaining higher cell viability compared with 2D culture.


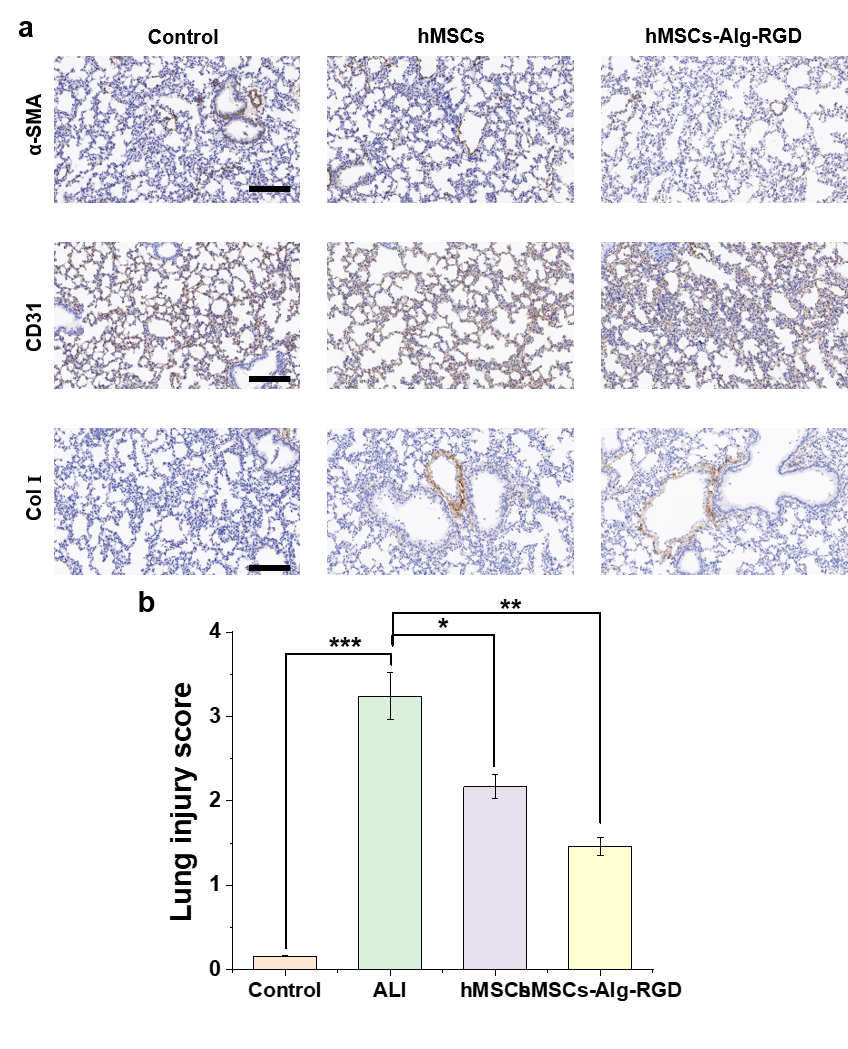


Figure S5. (a) Representative immunohistochemistry images of α-SMA, CD31, and Col Ⅰ in lung tissues after different treatments. (b) Lung injury scores across groups. The hMSCs-Alg-RGD group exhibited reduced α-SMA and collagen deposition, improved vascular integrity (CD31), and significantly lower histological scores compared with control and injury groups. Scale bar, 100 μm. *, p < 0.05; **, p < 0.01; ***, p < 0.001.


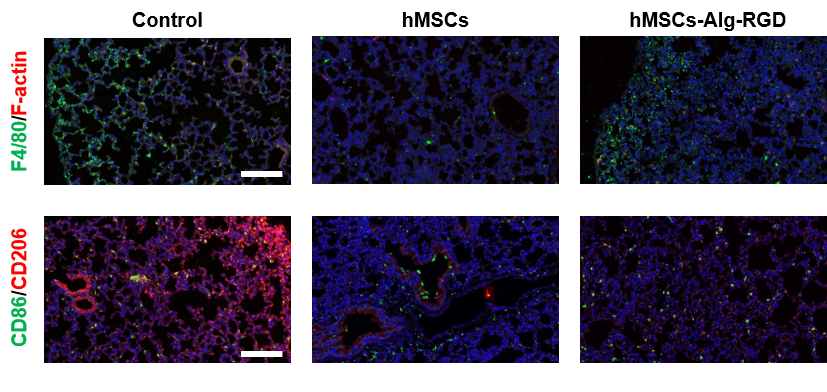


Figure S6. Immunohistochemistry analysis of macrophage infiltration and polarization in lung tissues. F4/80 staining revealed increased macrophage recruitment in the hMSCs-Alg-RGD group, while CD86 (M1 marker) decreased and CD206 (M2 marker) increased, indicating a shift toward reparative M2 polarization. Scale bar, 100 μm.


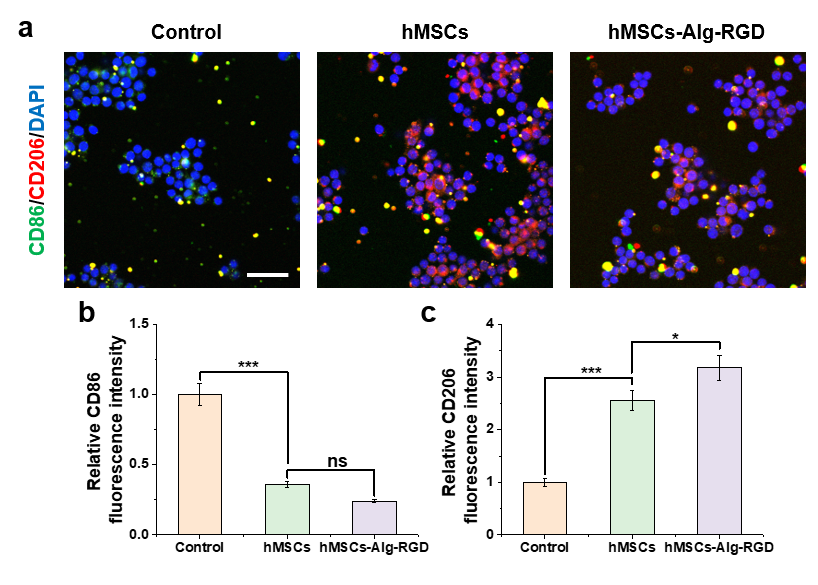


Figure S7. (a) Representative immunofluorescence images of macrophage polarization markers (green: CD86, red: CD206, blue: DAPI) after different treatments. (b–c) Quantification of CD86 and CD206 expression. hMSCs-Alg-RGD treatment reduced M1 and enhanced M2 markers. Scale bar, 100 μm. *, p < 0.05; **, p < 0.01; ***, p < 0.001.


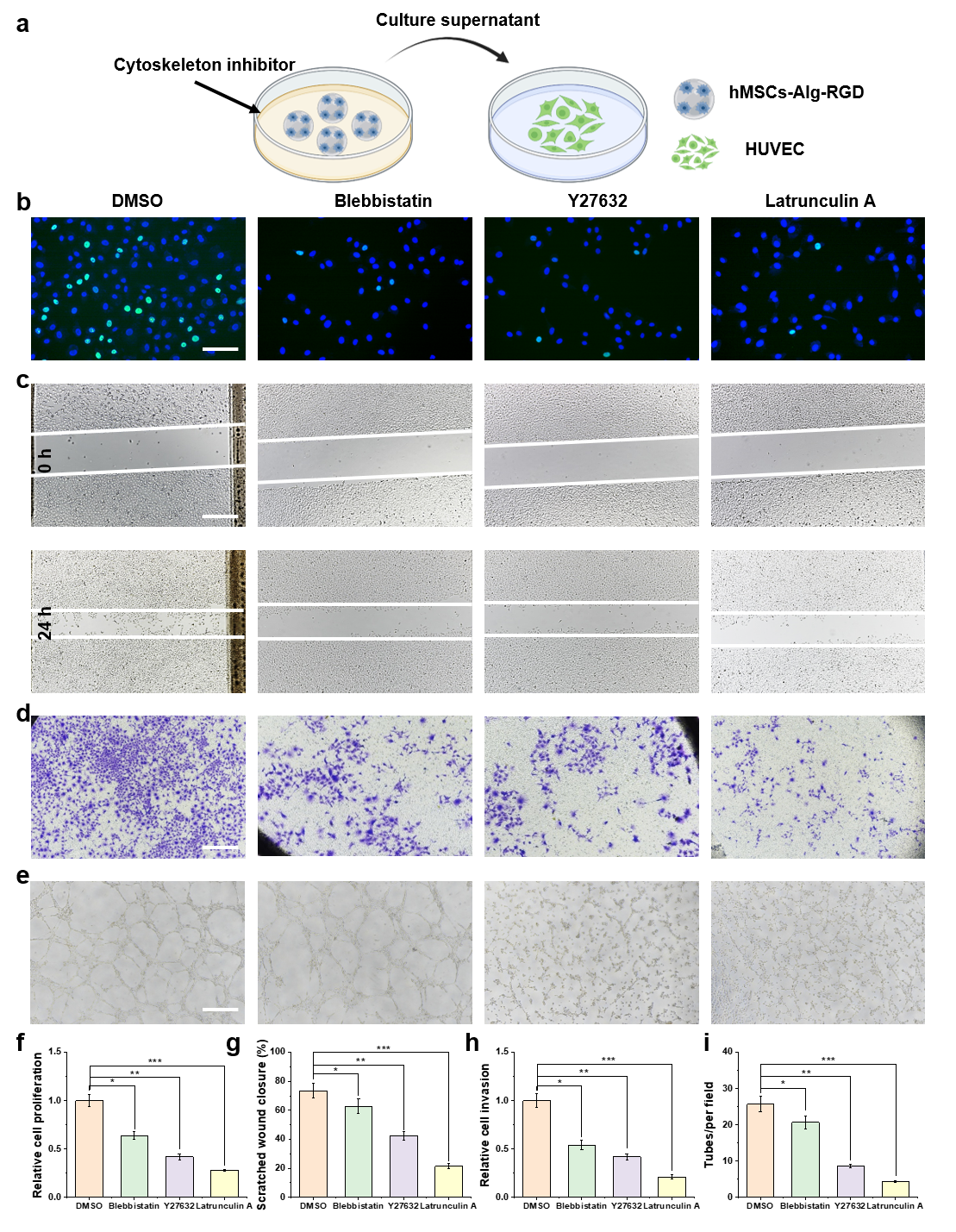


Figure S8. (a) Schematic analysis of the effect of cytoskeletal inhibitors on hMSCs-Alg-RGD regulatory functions. (b–e) Representative images showing proliferation (EdU staining, green: EdU, blue: DAPI), migration (scratch assay), invasion (Transwell assay), and tube formation (Matrigel assay) under different conditions. (f–i) Quantitative analysis of proliferation, migration, invasion, and tube formation. Inhibitors including Y-27632, Blebbistatin, and Latrunculin A attenuated the pro-angiogenic effects of hMSCs-Alg-RGD. Scale bar, 100 μm. *, p < 0.05; **, p < 0.01; ***, p < 0.001.


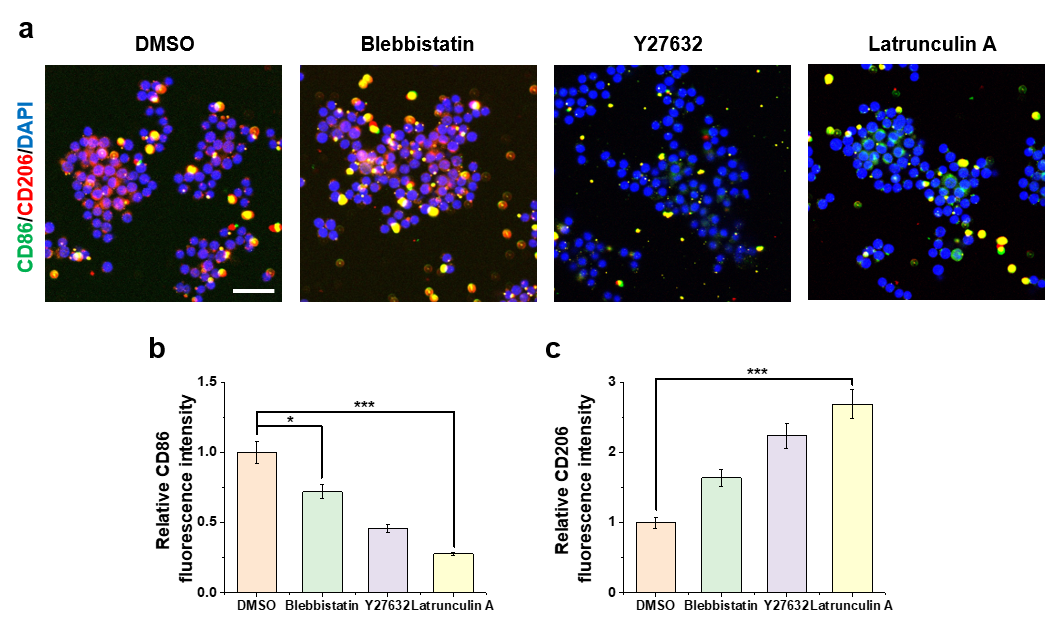


Figure S9. Analysis of cytoskeletal inhibition on hMSCs-Alg-RGD–mediated macrophage polarization. (a) Representative immunofluorescence images showing CD86 (green), CD206 (red), and DAPI (blue) after treatment with or without cytoskeletal inhibitors. (b–c) Quantitative analysis of CD86 and CD206 expression revealed that cytoskeletal inhibition partially reversed the M1→M2 shift induced by hMSCs-Alg-RGD. Scale bar, 100 μm. *, p < 0.05; **, p < 0.01; ***, p < 0.001.


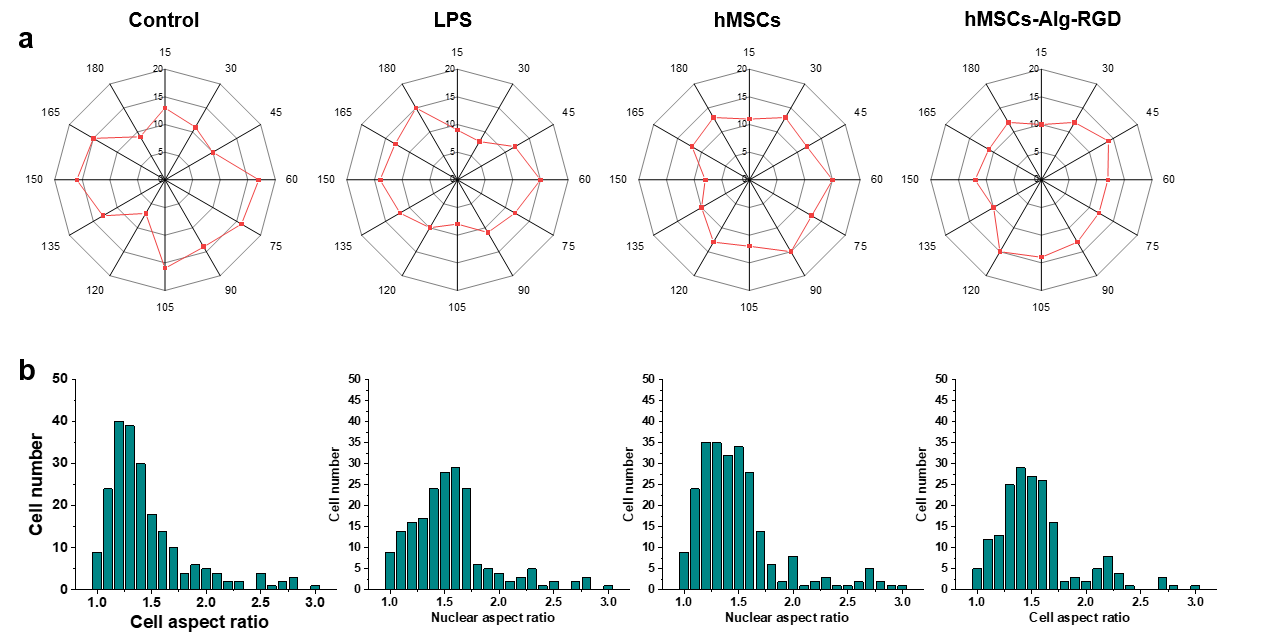


Figure S10. Morphological quantification of fibroblasts after different treatments. (a) Analysis of cellular orientation angles. (b) Statistical results of cell elongation index (length-to-width ratio), demonstrating reduced elongation in the hMSCs-Alg-RGD group, consistent with a low-tension cytoskeletal state.


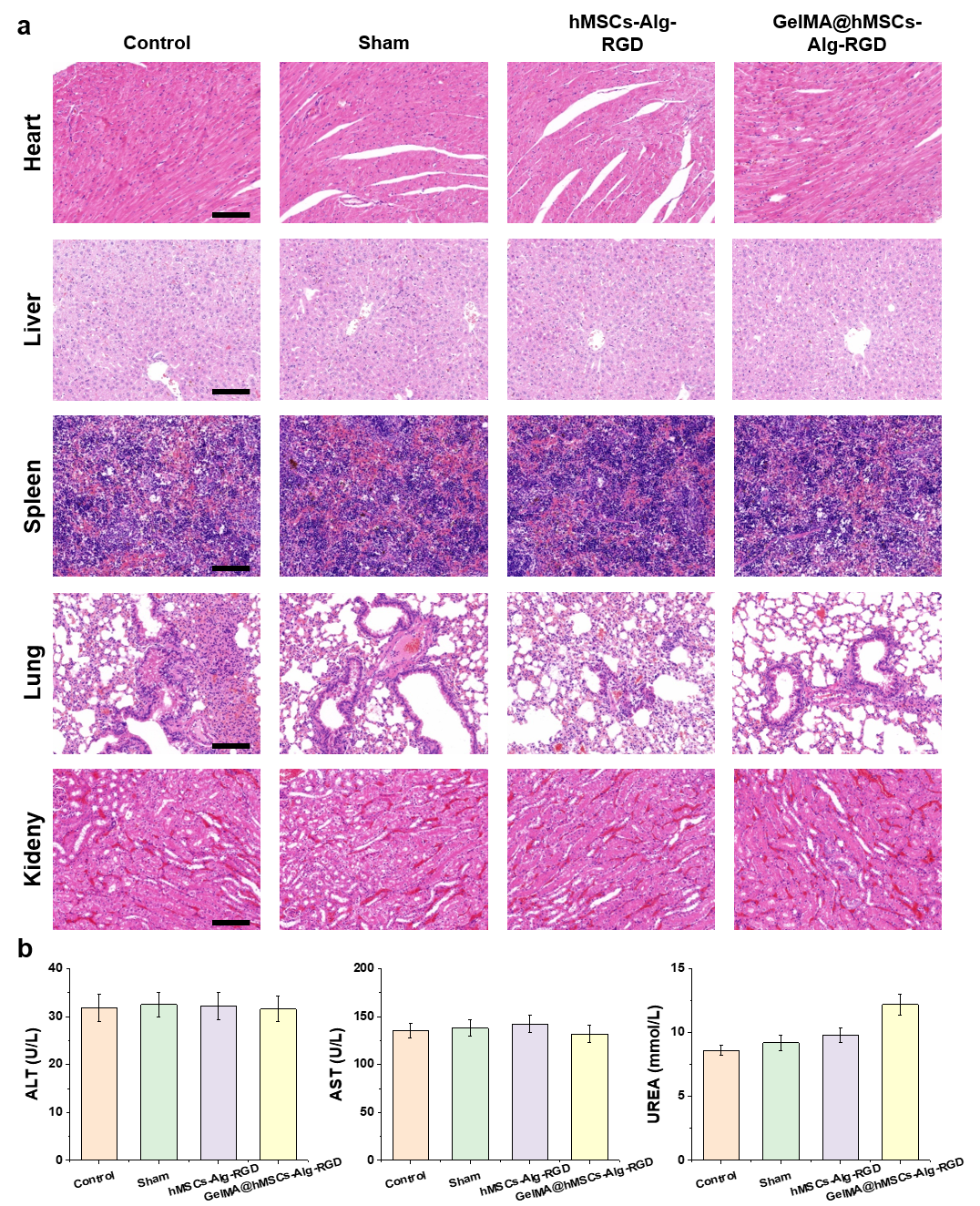


Figure S11. Biocompatibility Assessment of GelMA@hMSCs-Alg-RGD

(a) Representative H&E staining images of major organs (heart, liver, spleen, lung, and kidney) from mice at 28th with different materials.

(b) Serum biochemical parameters for liver and kidney function (ALT, AST, BUN, and creatinine) showing no significant abnormalities among groups, confirming the biosafety of GelMA@hMSCs-Alg-RGD.

Scale bar, 100 μm.


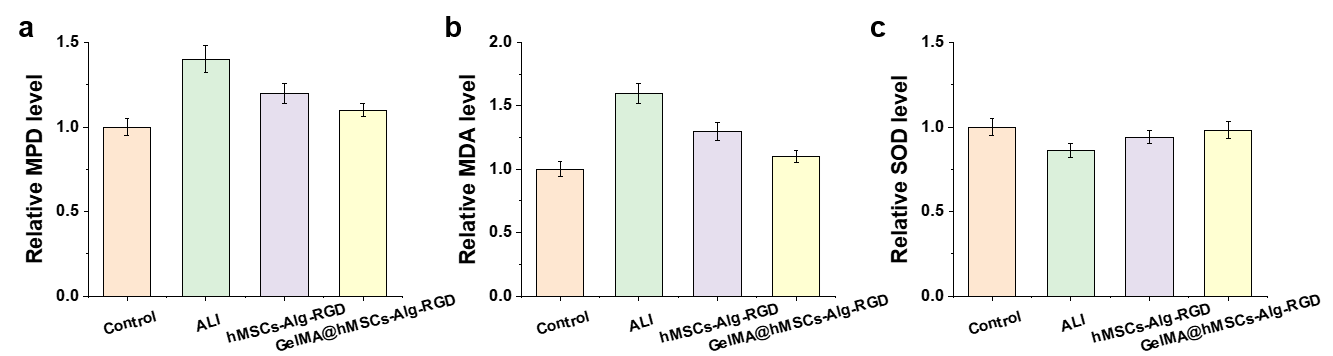


Figure S12. Oxidative stress markers in lung tissue homogenates at Day 28 after LPS-induced ALI and treatments.
(a-c) Quantification of myeloperoxidase (MPO) activity, malondialdehyde (MDA) content, and superoxide dismutase (SOD) activity in lung tissue homogenates collected on Day 28 from the four groups. n = 5 mice per group.


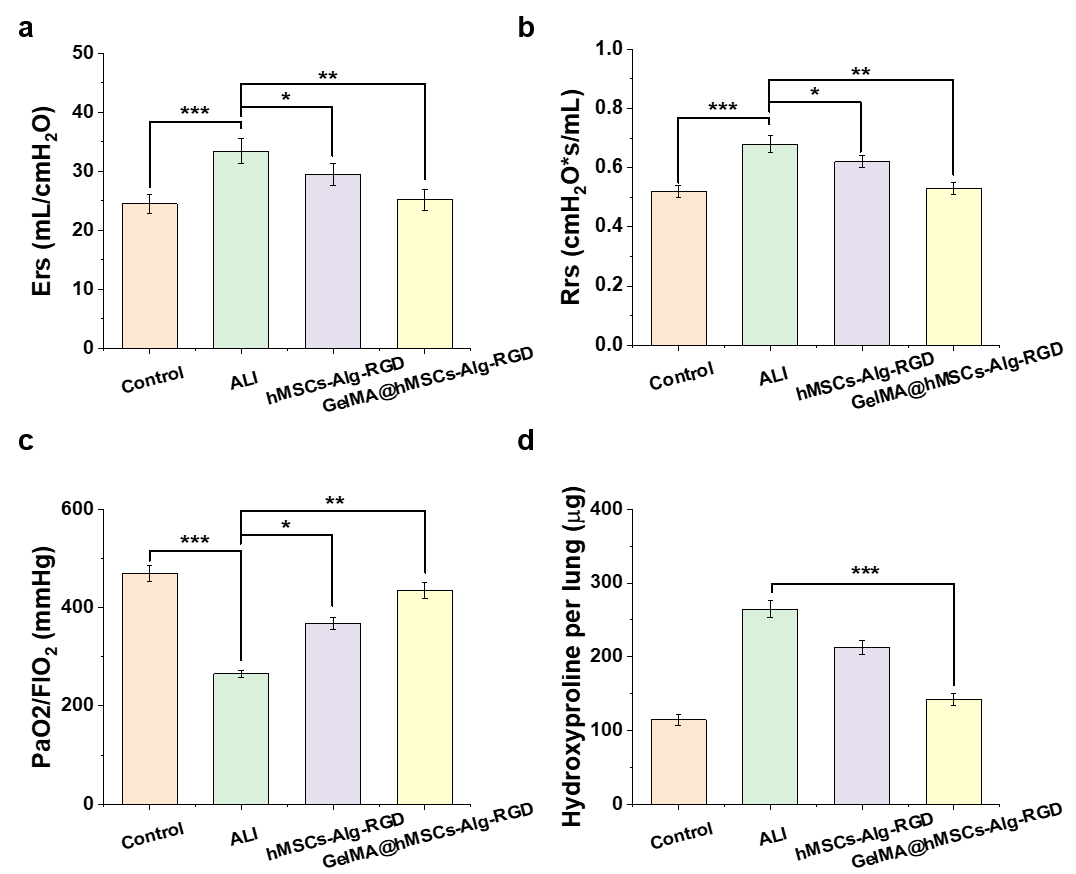


Figure S13. Short-term and long-term functional outcomes after LPS-induced ALI.
(a) Respiratory system elastance (Ers) measured by small-animal respiratory mechanics testing (flexiVent FX system) at the acute time point after LPS challenge.
(b) Respiratory system resistance (Rrs) measured in the same animals and time point as in (a).
(c) Oxygenation index (PaO₂/FiO₂, P/F) determined by arterial blood gas analysis at the acute time point.
(d) Lung hydroxyproline content quantified on day 28 as an indicator of collagen deposition/fibrotic remodeling.

*P < 0.05, **P < 0.01, ***P < 0.001.


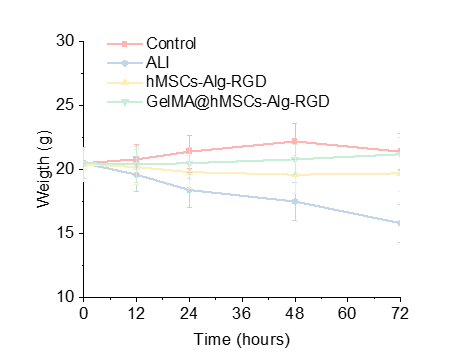


Figure S14. Body weight monitoring during the acute ALI study.


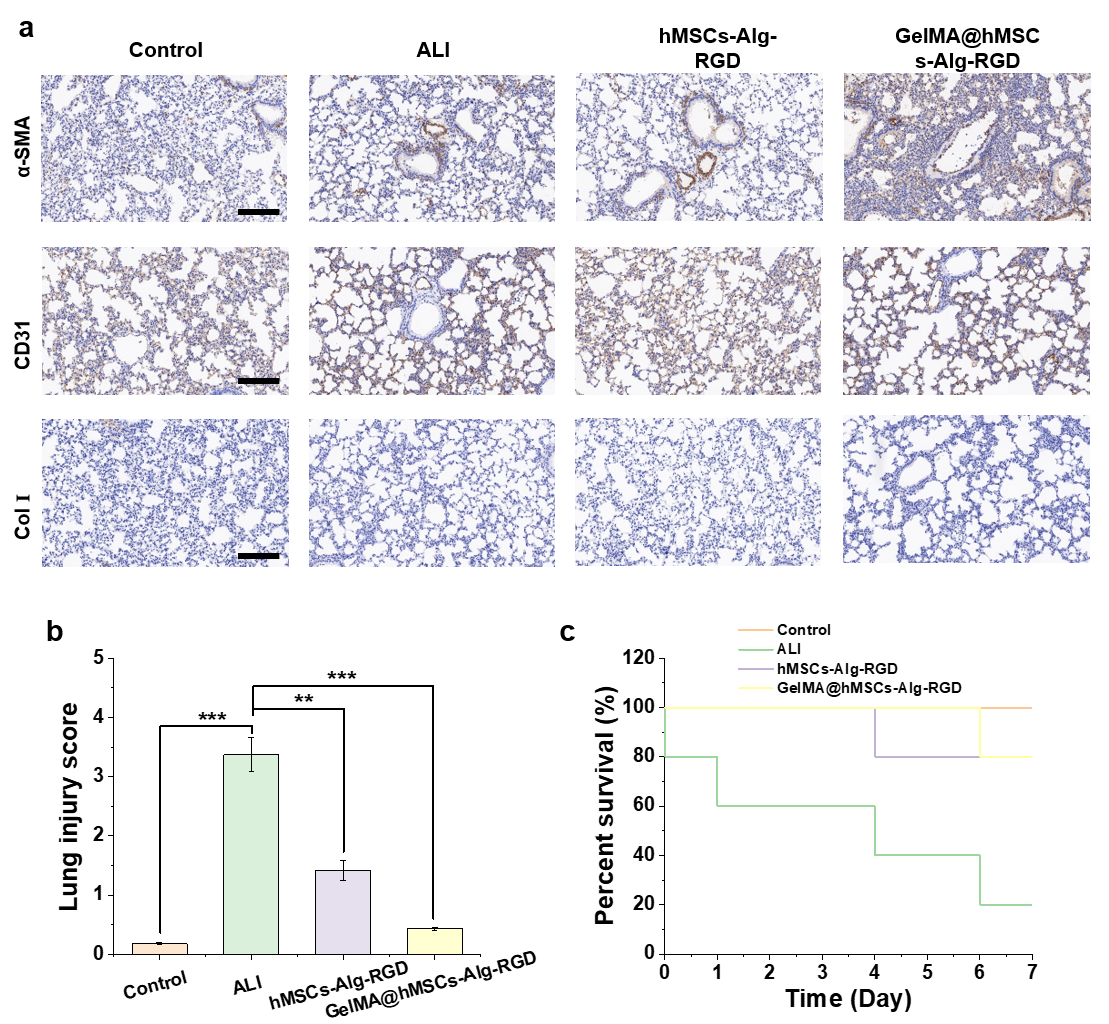


Figure S15. Therapeutic Efficacy of GelMA@hMSCs-Alg-RGD in ALI Model

(a) Representative immunohistochemical staining of α-SMA, CD31, and collagen I in lung tissues from different groups after treatment.

(b) Quantitative lung injury scores of each group, showing significant histological improvement in the GelMA@hMSCs-Alg-RGD group.

(c) Kaplan–Meier survival curves of mice in different groups during the treatment period, demonstrating enhanced survival in the GelMA@hMSCs-Alg-RGD group.

Scale bar, 100 μm. *, p < 0.05; **, p < 0.01; ***, p < 0.001.


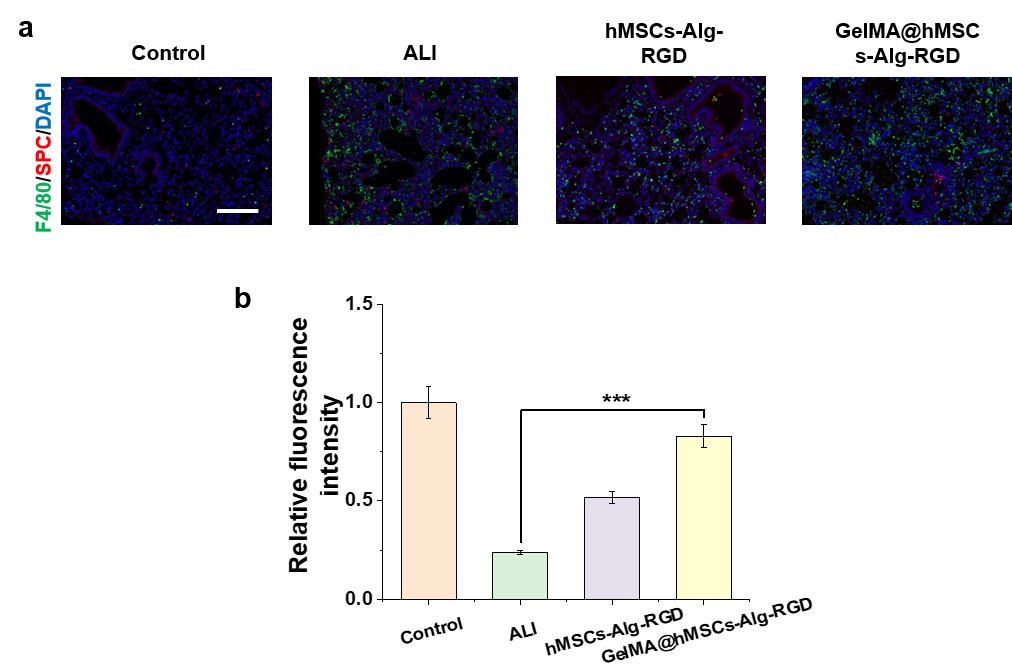


Figure S16. Additional validation of macrophage polarization in lung tissue.

(a) Representative dual-color immunofluorescence of F4/80 and SPC with DAPI in Control, ALI, hMSCs-Alg-RGD, and GelMA-DA@hMSCs-Alg-RGD groups;

(b) Quantitative analysis of fluorescence intensity.

Scale bar, 100 μm. *, p < 0.05; **, p < 0.01; ***, p < 0.001.
