# Supplementary figures and images for: A Mechanotransduction-Aware Strategy for Enhancing MSC Potency via 3D Culture and Localized Delivery
Source: Cyborg Bionic Syst. 2026 Mar 24;7:0552. doi: 10.34133/cbsystems.0552 (PMC13009532; doi:10.34133/cbsystems.0552)

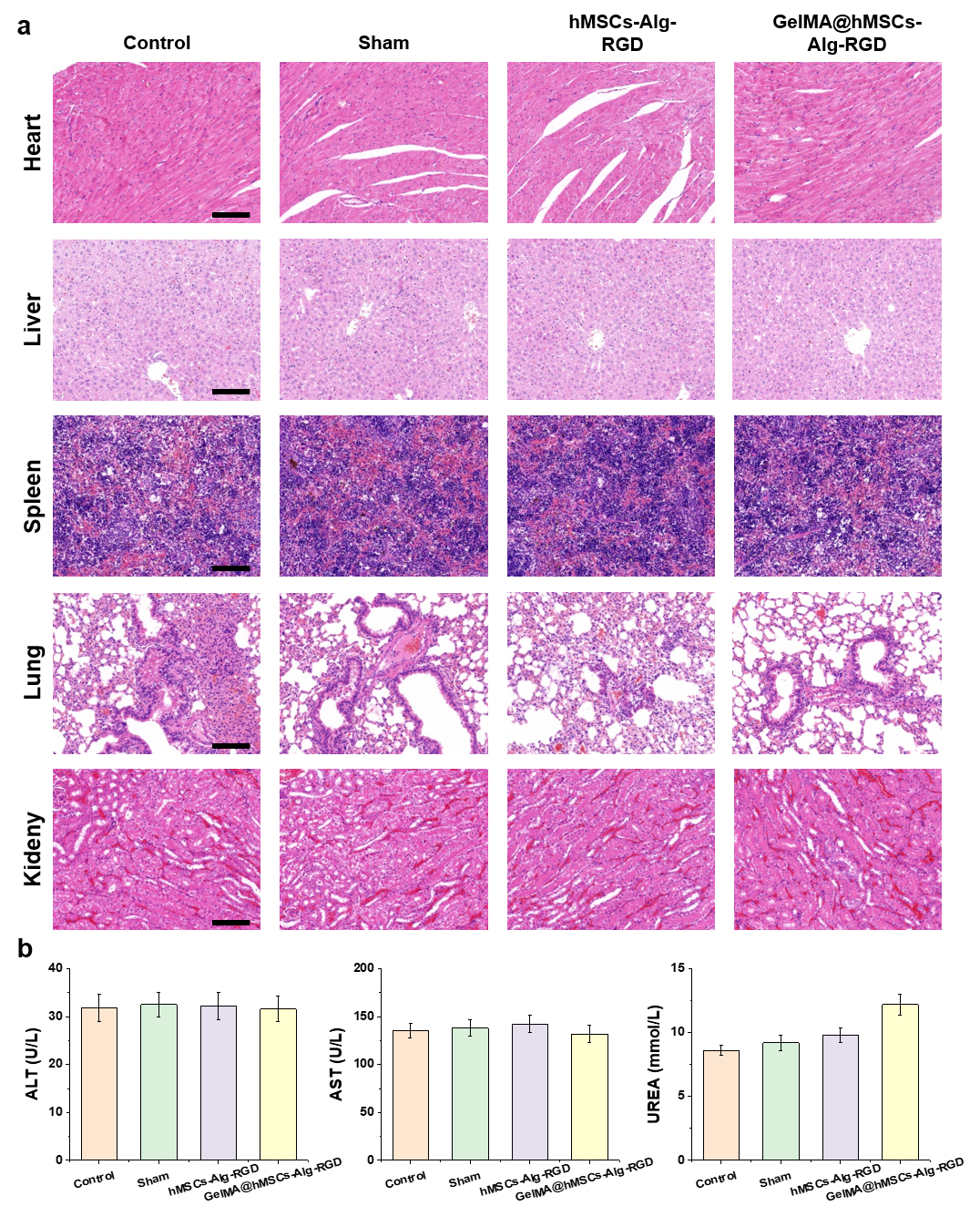

Supplement: Supplementary 1 — Figs. S1 to S16 [file cbsystems.0552.f1.zip › Revised Figure S11.tif]

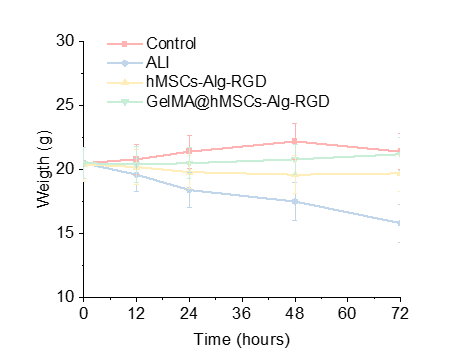

Supplement: Supplementary 1 — Figs. S1 to S16 [file cbsystems.0552.f1.zip › Revised Figure S14.tif]

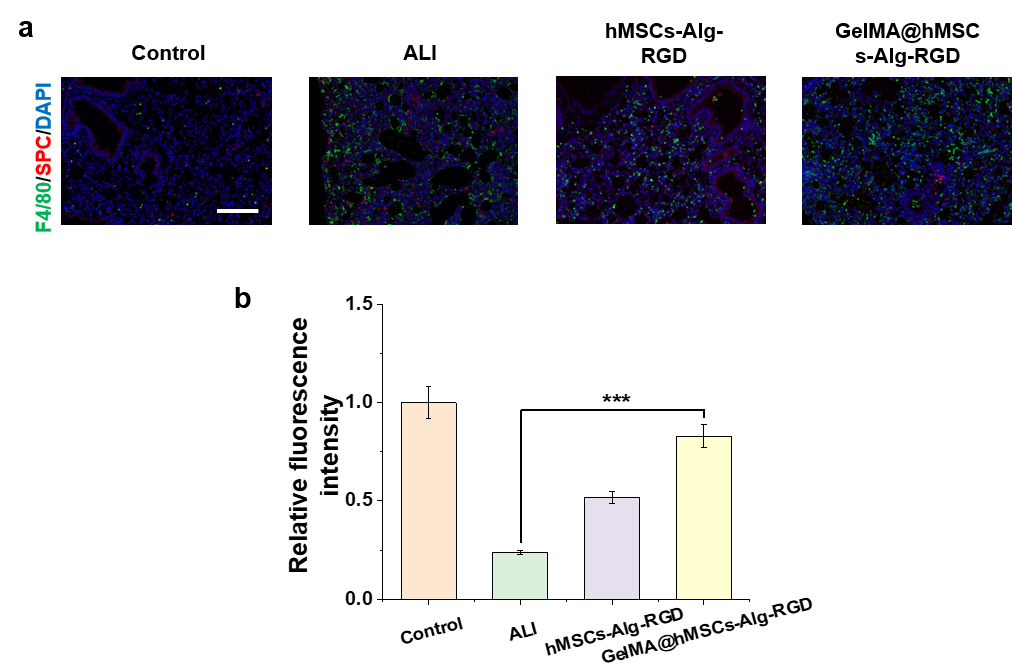

Supplement: Supplementary 1 — Figs. S1 to S16 [file cbsystems.0552.f1.zip › Revised Figure S16.tif]

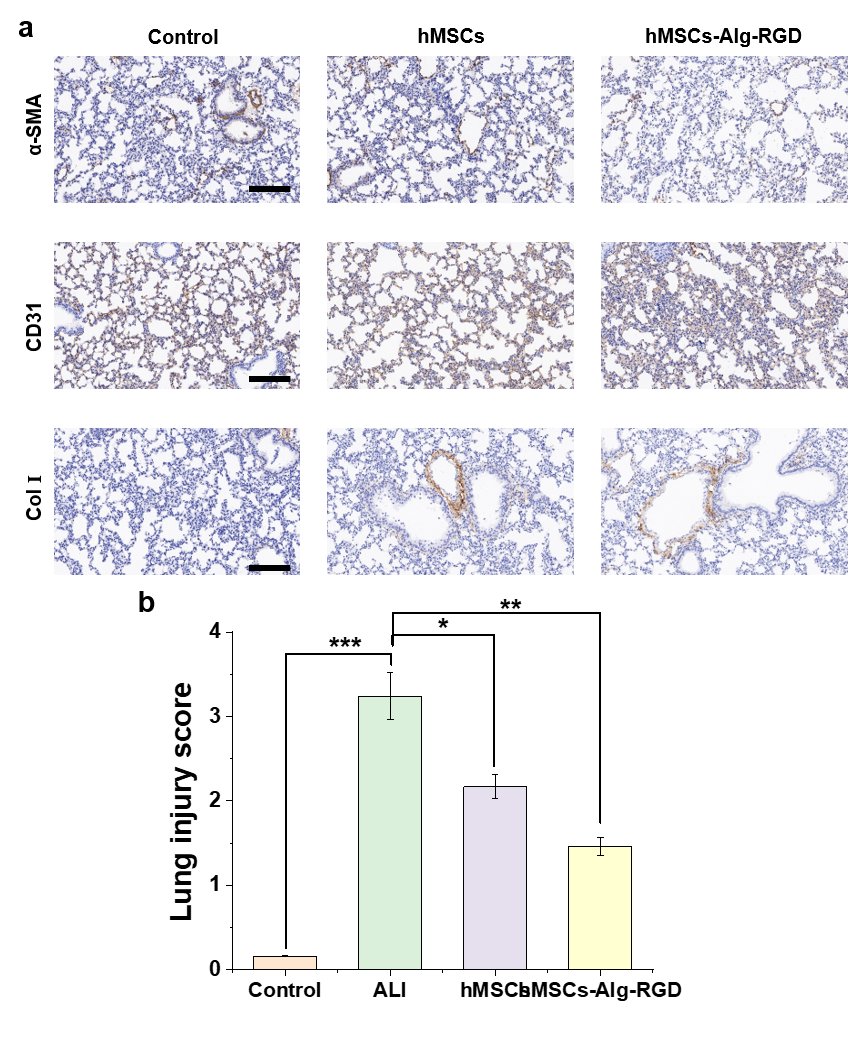

Supplement: Supplementary 1 — Figs. S1 to S16 [file cbsystems.0552.f1.zip › Revised Figure S5.tif]

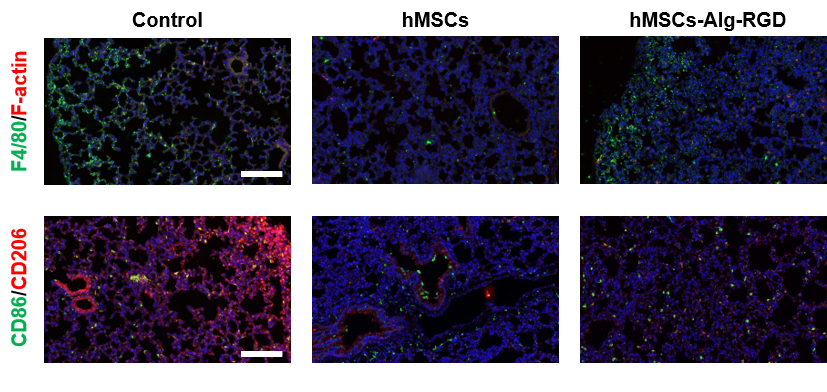

Supplement: Supplementary 1 — Figs. S1 to S16 [file cbsystems.0552.f1.zip › Revised Figure S6.tif]

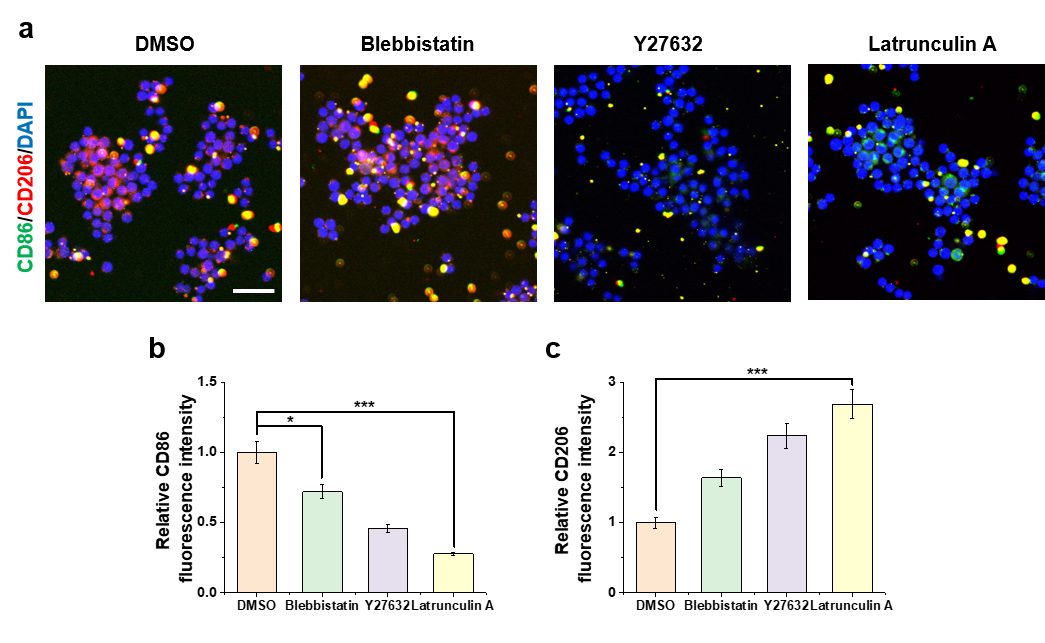

Supplement: Supplementary 1 — Figs. S1 to S16 [file cbsystems.0552.f1.zip › Revised Figure S9.tif]

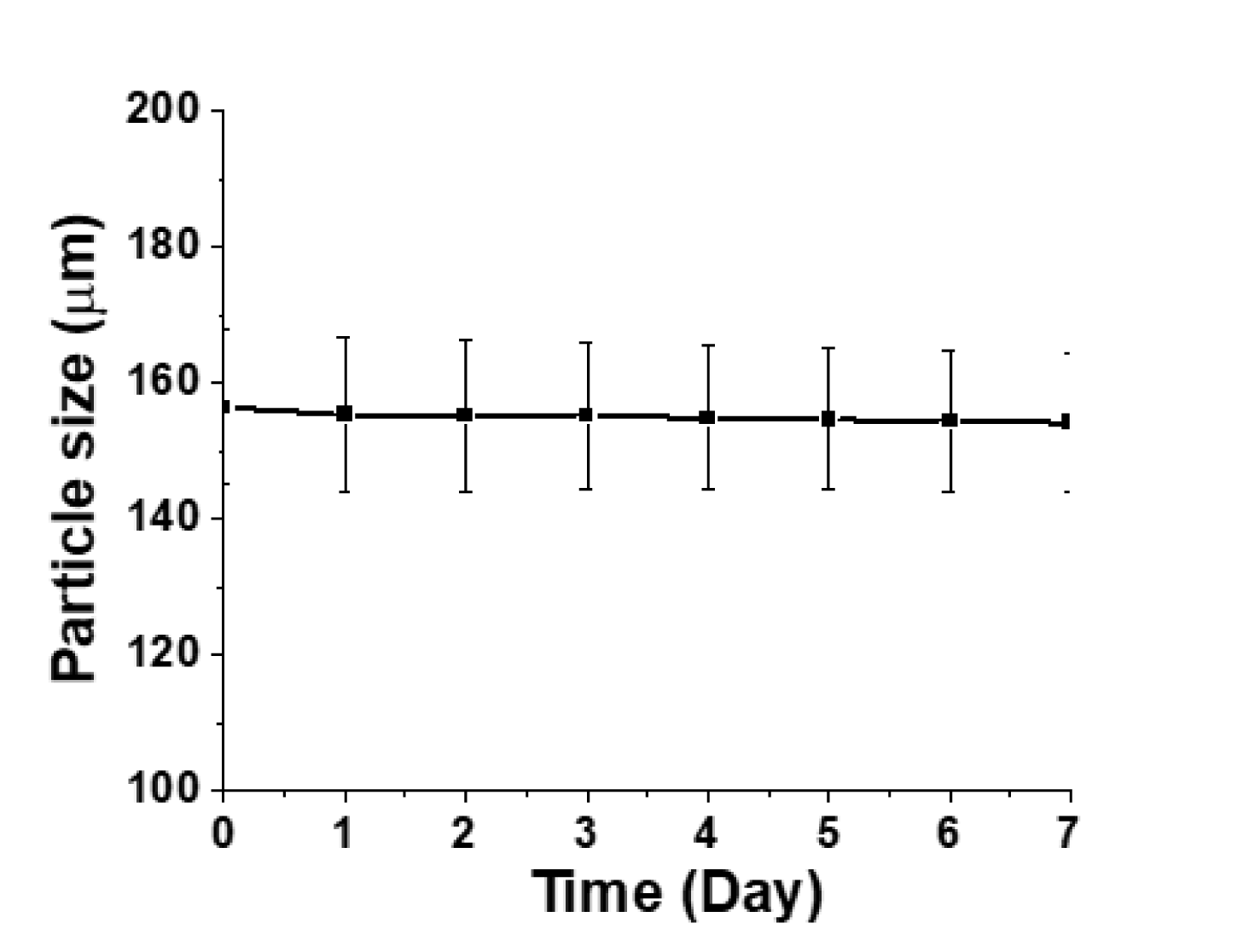

Supplement: Supplementary 1 — Figs. S1 to S16 [file cbsystems.0552.f1.zip › Revised S1.tif]

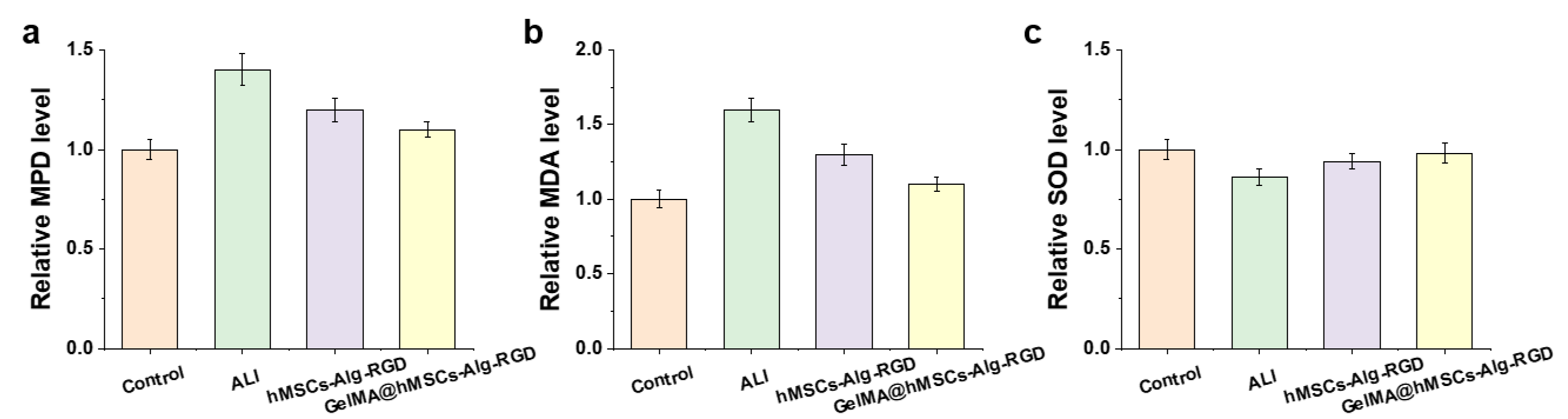

Supplement: Supplementary 1 — Figs. S1 to S16 [file cbsystems.0552.f1.zip › Revised S12.tif]

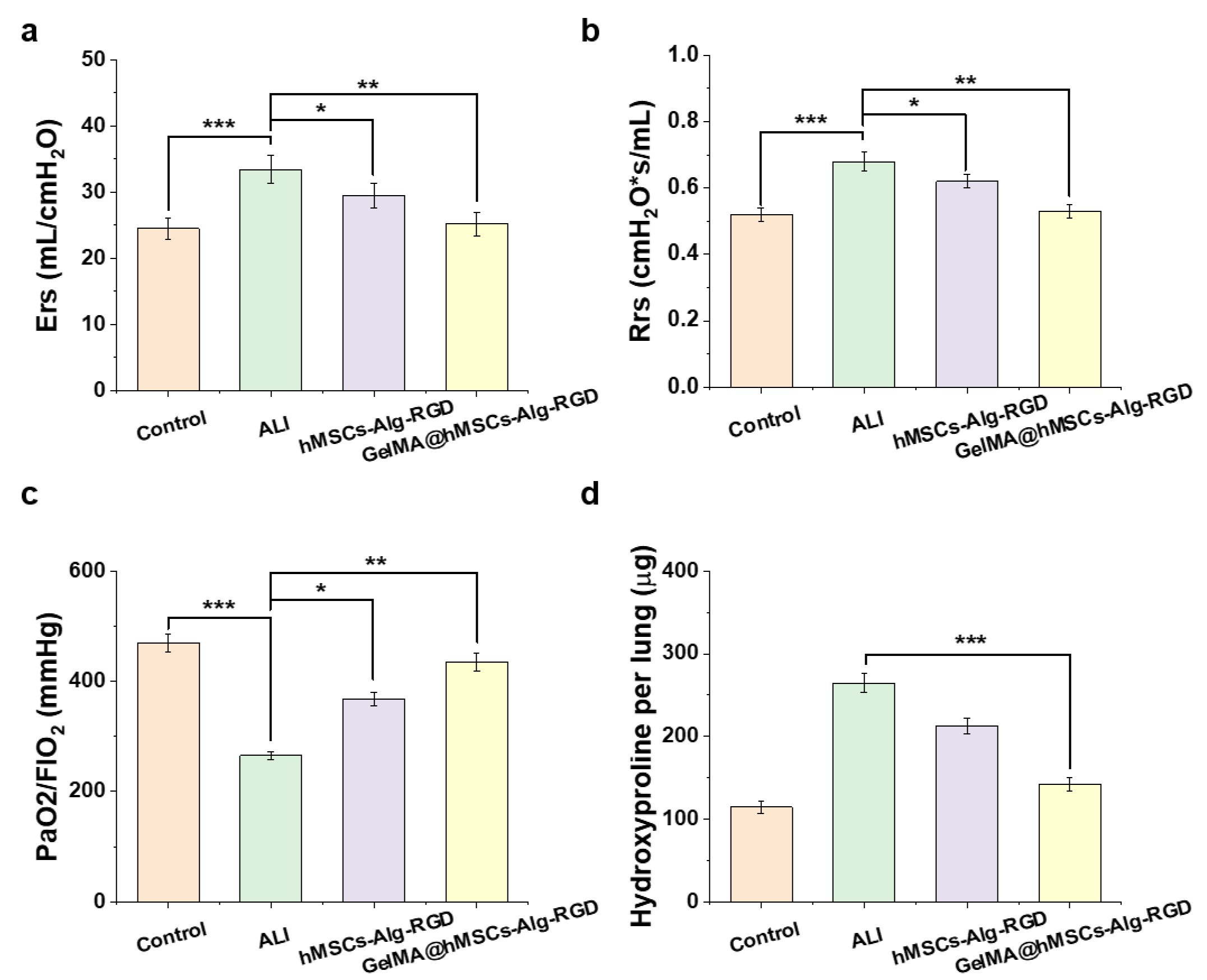

Supplement: Supplementary 1 — Figs. S1 to S16 [file cbsystems.0552.f1.zip › Revised S13.tif]

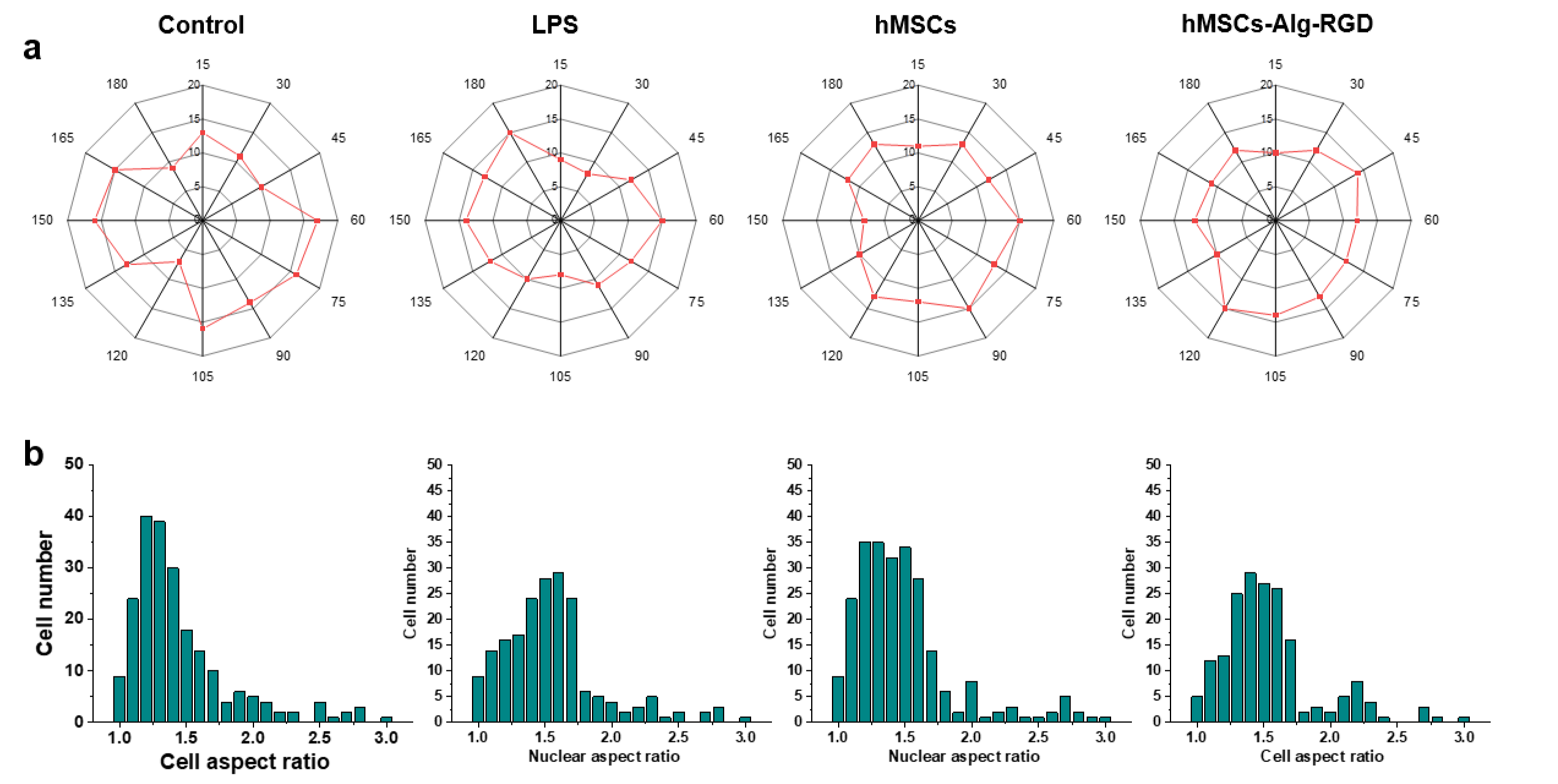

Supplement: Supplementary 1 — Figs. S1 to S16 [file cbsystems.0552.f1.zip › S10.tif]

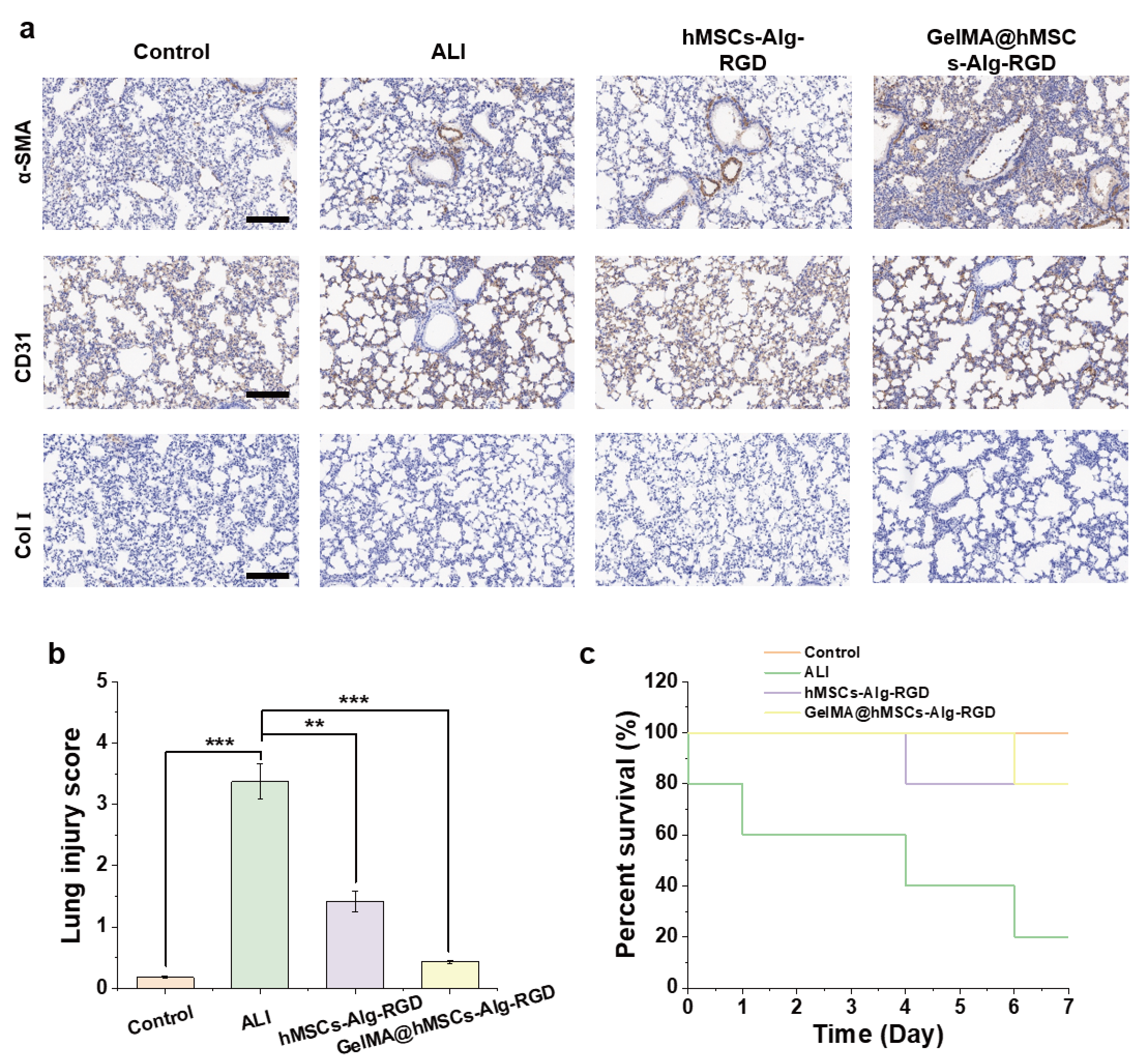

Supplement: Supplementary 1 — Figs. S1 to S16 [file cbsystems.0552.f1.zip › S15.tif]

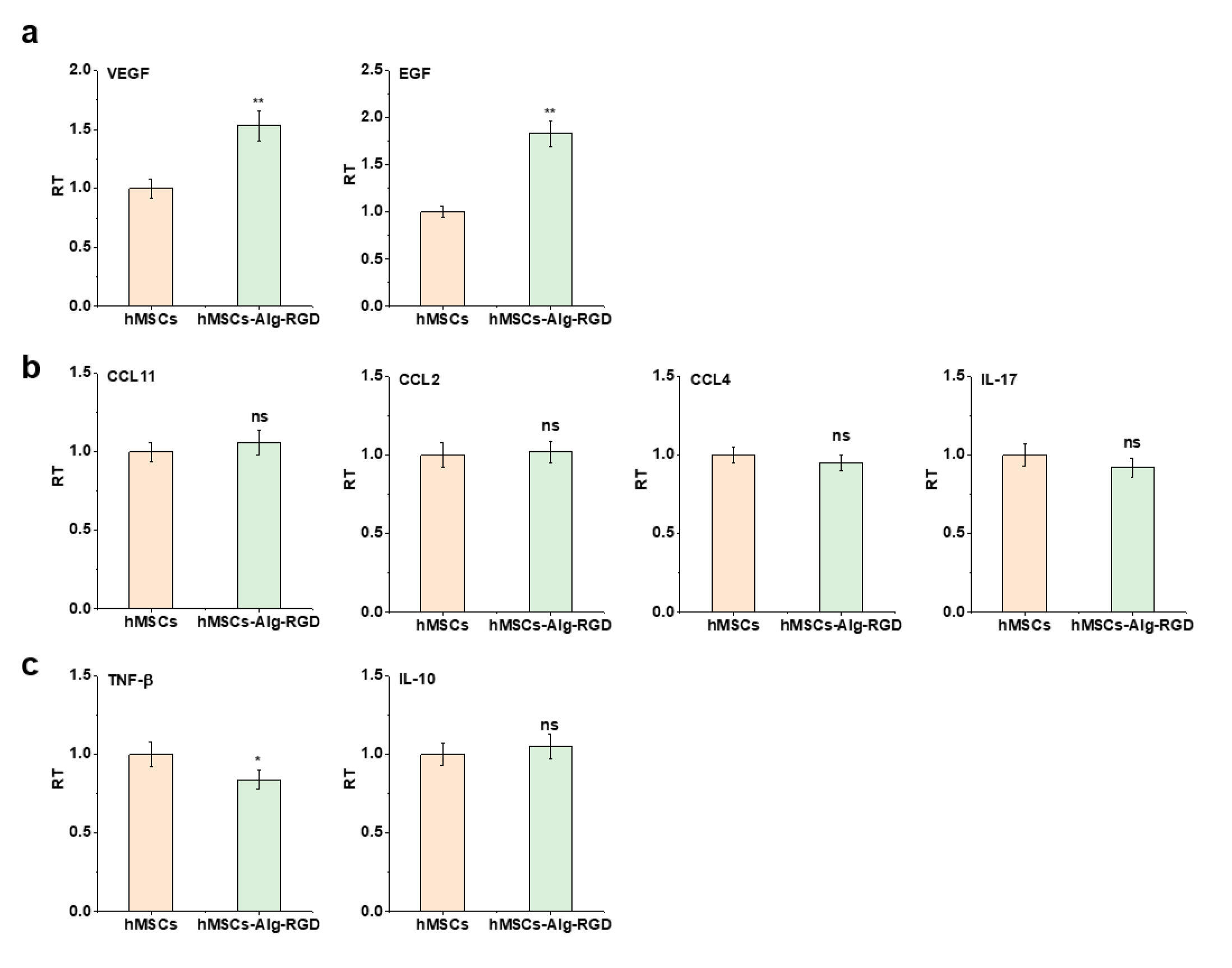

Supplement: Supplementary 1 — Figs. S1 to S16 [file cbsystems.0552.f1.zip › S2.tif]

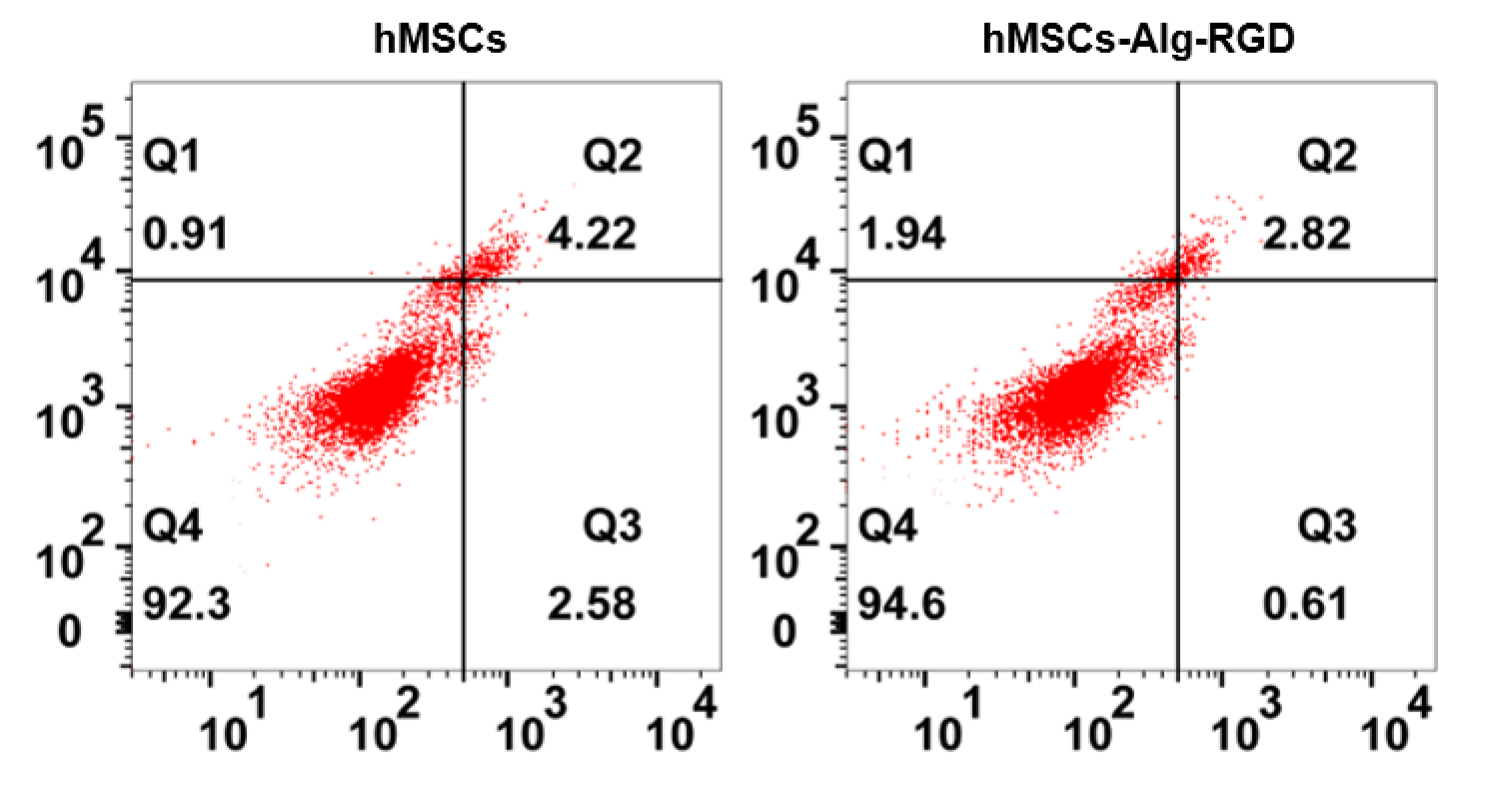

Supplement: Supplementary 1 — Figs. S1 to S16 [file cbsystems.0552.f1.zip › S3.tif]

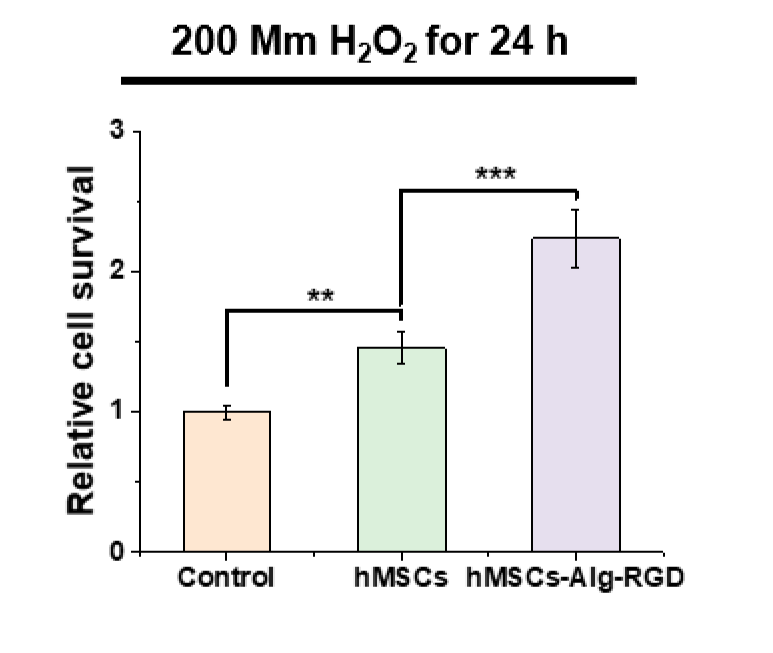

Supplement: Supplementary 1 — Figs. S1 to S16 [file cbsystems.0552.f1.zip › S4.tif]

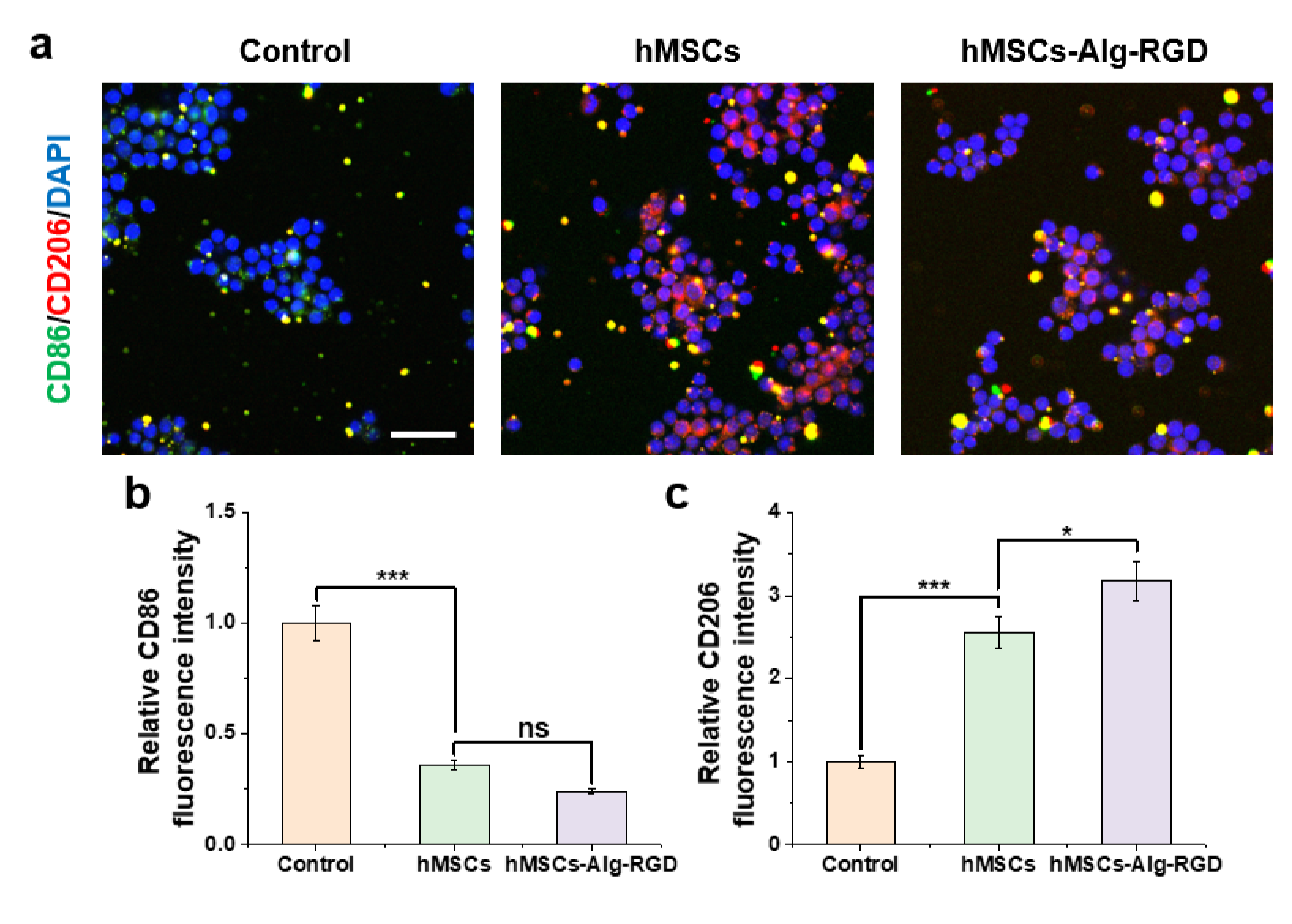

Supplement: Supplementary 1 — Figs. S1 to S16 [file cbsystems.0552.f1.zip › S7.tif]

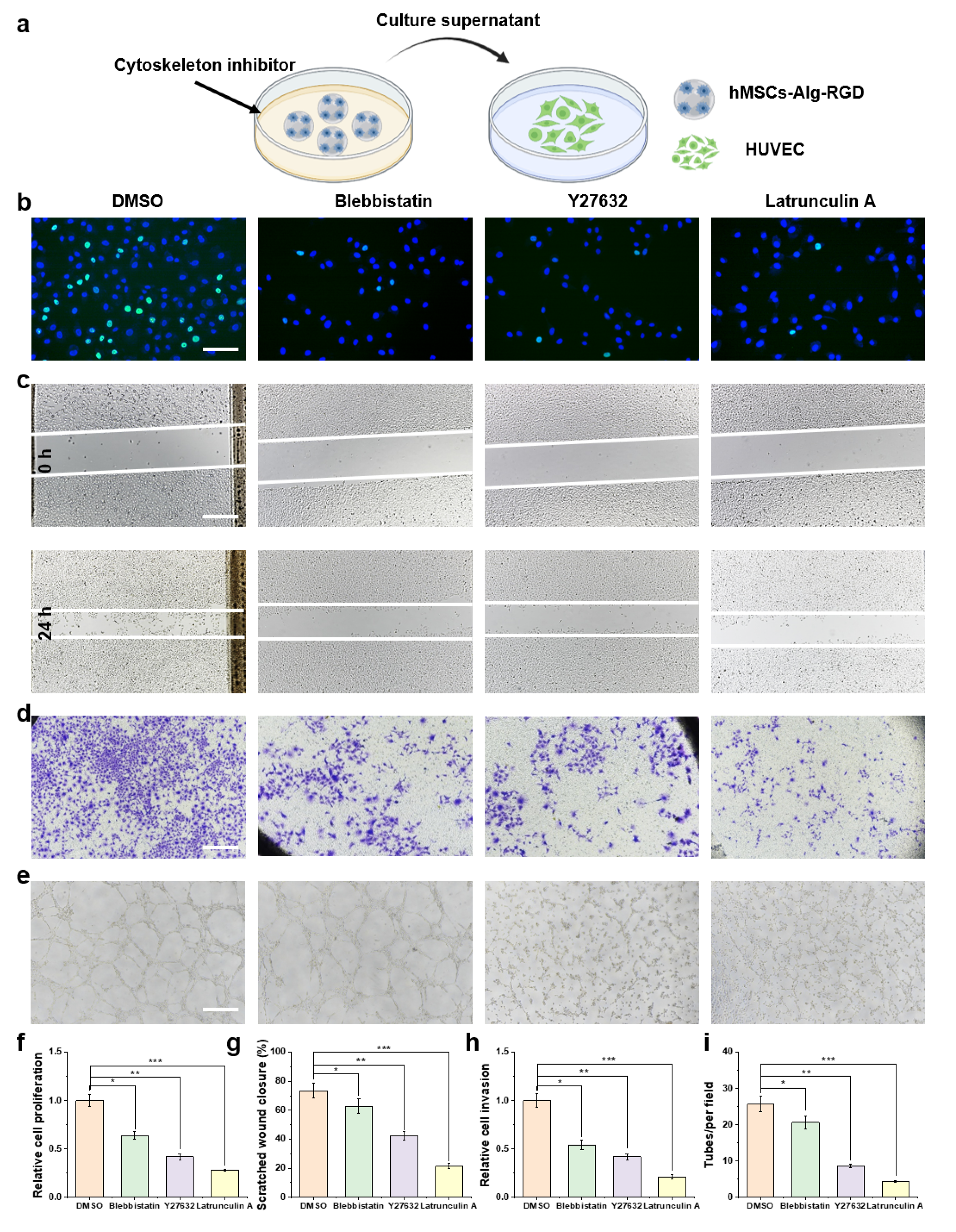

Supplement: Supplementary 1 — Figs. S1 to S16 [file cbsystems.0552.f1.zip › S8.tif]
